# Genome-wide analysis of WRKY gene family in *Cucumis sativus*

**DOI:** 10.1186/1471-2164-12-471

**Published:** 2011-09-28

**Authors:** Jian Ling, Weijie Jiang, Ying Zhang, Hongjun Yu, Zhenchuan Mao, Xingfang Gu, Sanwen Huang, Bingyan Xie

**Affiliations:** 1 Institute of Vegetables and Flowers, Chinese Academy of Agricultural Sciences, 12 Zhongguancun South Street, Beijing, 100081 China

## Abstract

**Background:**

WRKY proteins are a large family of transcriptional regulators in higher plant. They are involved in many biological processes, such as plant development, metabolism, and responses to biotic and abiotic stresses. Prior to the present study, only one full-length cucumber WRKY protein had been reported. The recent publication of the draft genome sequence of cucumber allowed us to conduct a genome-wide search for cucumber WRKY proteins, and to compare these positively identified proteins with their homologs in model plants, such as *Arabidopsis*.

**Results:**

We identified a total of 55 WRKY genes in the cucumber genome. According to structural features of their encoded proteins, the cucumber WRKY (*CsWRKY*) genes were classified into three groups (group 1-3). Analysis of expression profiles of *CsWRKY *genes indicated that 48 WRKY genes display differential expression either in their transcript abundance or in their expression patterns under normal growth conditions, and 23 WRKY genes were differentially expressed in response to at least one abiotic stresses (cold, drought or salinity). The expression profile of stress-inducible *CsWRKY *genes were correlated with those of their putative *Arabidopsis WRKY (AtWRKY) *orthologs, except for the group 3 WRKY genes. Interestingly, duplicated group 3 *AtWRKY *genes appear to have been under positive selection pressure during evolution. In contrast, there was no evidence of recent gene duplication or positive selection pressure among *CsWRKY *group 3 genes, which may have led to the expressional divergence of group 3 orthologs.

**Conclusions:**

Fifty-five WRKY genes were identified in cucumber and the structure of their encoded proteins, their expression, and their evolution were examined. Considering that there has been extensive expansion of group 3 WRKY genes in angiosperms, the occurrence of different evolutionary events could explain the functional divergence of these genes.

## Background

Transcription factors exhibit sequence-specific DNA-binding and are capable of activating or repressing transcription of downstream target genes. In plants, WRKY proteins constitute a large family of transcription factors that are involved in various physiological processes. Proteins in this family contain at least one highly conserved signature domain of about 60 amino acid residues, which includes the conserved WRKYGQK sequence followed by a zinc finger motif, located in the C-terminal region [1]. The WRKY domain facilitates binding of the proteins to the W box or the SURE (sugar-responsive cis-element) in the promoter regions of target genes [2,3]. As deduced from nuclear magnetic resonance (NMR) analysis of the C-terminal WRKY domain of *Arabidopsis *WRKY4 (*AtWRKY4*), the conserved WRKYGQK sequence of WRKY domains is directly involved in DNA binding [4]. WRKY proteins can be classified into three groups (1, 2 and 3) based on the number of WRKY domains and the pattern of the zinc-finger motif. Group 1 proteins typically contain two WRKY domains including a C2H2 motif. Group 2 proteins have a single WRKY domain and a C2H2 zinc-finger motif and can be further divided into five subgroups (2a-2e) based on the phylogeny of the WRKY domains. Group 3 proteins also have a single WRKY domain, but their zinc-finger-like motif is C2-H-C [1].

Since the cloning of the first cDNA encoding a WRKY protein, *SPF1 *from sweet potato [5], a large number of WRKY proteins have been experimentally identified from several plant species [6-17], and have been shown to be involved in various physiological processes under normal growth conditions and under various stress condition [18]. It has been well documented that WRKY proteins play a key role in plant defense against various biotic stresses including bacterial, fungal and viral pathogens [19-27]. They also play important regulatory roles in developmental processes, such as trichome initiation [28], embryo morphogenesis [29], senescence [30], and some signal transduction processes mediated by plant hormones such as gibberellic acid [31], abscisic acid [32,33] or salicylic acid [34]. There is also accumulating evidence that WRKY proteins are involved in responses to various abiotic stresses. In *Arabidopsis*, microarray analyses have revealed that some of the WRKY transcripts are strongly regulated in response to various abiotic stresses, such as salinity, drought and cold [35-37]. In rice, under abiotic stresses (cold, drought and salinity) or various phytohormone treatments, 54 WRKY genes showed significant differences in their transcript abundance [18]. In barley, a WRKY gene, *Hv-WRKY38*, is expressed in response to cold and drought stress response [38] while in soybean at least nine WRKY genes are found to be differentially expressed under abiotic stress [15].

Because of their extensive involvement in various physiological processes, it is likely that the WRKY family in angiosperms has expanded greatly during evolution. There are at least 72 WRKY family members in *Arabidopsis *[1] and at least 109 in rice [17]. Gene duplication events have played a critical role in the expansion of WRKY genes. For example, in rice, 80% of WRKY genes loci are located in duplicated regions [18]. Gene duplication events can lead to the generation of new WRKY genes. It is worth noting that the three groups of WRKY genes appeared at different times during evolution. Most members of groups 1 and 2 appear to have arisen before the divergence of the monocots and dicots, while group 3 WRKY genes seem to have had a relative later origin [17]. In addition, a recent study showed that expression divergence had occurred among duplicated WRKY genes [18]. However, the reasons for expression divergence among duplicated WRKY genes remain unclear.

Cucumber is not only an economically important cultivated plant, but also a model system for studies on sex determination and plant vascular biology [39]. A draft of the *Cucumis sativus var. sativus L*. genome sequence was reported recently [40]. In this study, we searched this genome sequence to identify the WRKY genes of cucumber (*CsWRKY*). Then, we analyzed the expression of the identified *CsWRKY *genes under normal growth conditions and under various abiotic stresses conditions. We compared the structure of the encoded proteins and the expression profiles of C*sWRKY *genes with those of their putative homologs in *Arabidopsis thaliana *WRKY (*AtWRKY*) genes, and found that there were notable difference between group 3 WRKY genes of *Arabidopsis *and cucumber. The evolutionary analysis of group 3 WRKY genes indicated that, unlike cucumber, the recent duplicated WRKY genes of *Arabidopsis *have been under positive selection pressure. This may explain the expression divergence of their orthologs. These studies will be useful for understanding the role of WRKY genes in plant responses to abiotic stresses. In addition, these results provide information about the relationship between evolution and functional divergence of the WRKY family.

## Results

### Identification of WRKY family in cucumber

**Figure 1:**
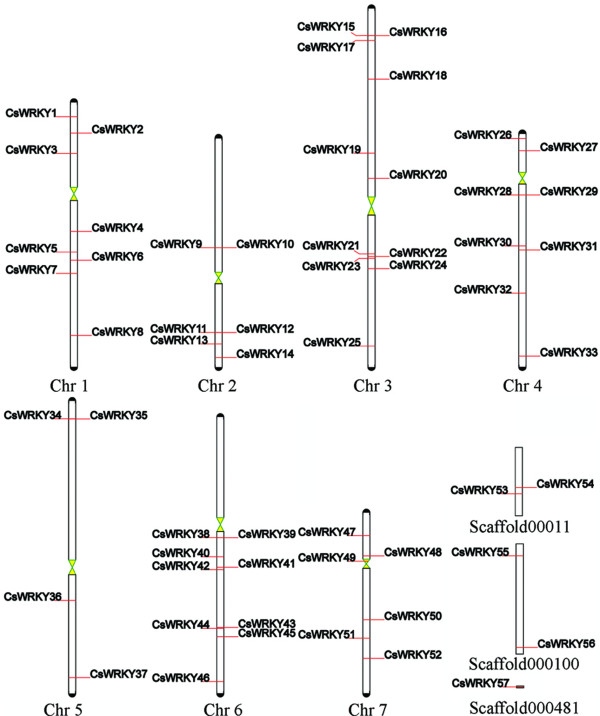
**Mapping of the WRKY gene family on *Cucumis sativus *L. chromosomes**. The size of a chromosome is indicated by its relative length. To simplify the presentation, we renamed the putative WRKY genes from *CsWKRY1 *to *CsWRKY52 *based on their order on the chromosomes. Five putative WRKY genes could not be localized on a specific chromosome, so we renamed them from *CsWRKY53 *to *CsWRKY57 *according to their raw scores in a search of cucumber WRKY proteins with the Hmmsearch program.

A total of 57 genes in the cucumber genome were identified as possible members of the WRKY superfamily and they encoded 57 WRKY proteins. Among these proteins, annotation of eight proteins revealed that they have two complete WRKY domains each. A total of 52 WRKY genes could be mapped on the chromosomes and were renamed from *CsWRKY1 *to *CsWRKY52 *based on their order on the chromosomes, from chromosomes 1 to 7 (Figure 1). Five WRKY genes (*Csa018657, Csa018622, Csa018069, Csa018094 *and *Csa022995*) that could not be conclusively mapped to any chromosome were renamed *CsWRKY53*-*CsWRKY57 *respectively. In addition, the nucleotide sequence of *Csa026380 *was completely identical to that of *Csa014665*, therefore; the latter was eliminated from this study.

Next, to establish whether these WRKY genes are expressed, we screened the cucumber EST database in NCBI. Twenty-seven putative WRKY genes matched at least one EST hits (Table 1). We cloned and sequenced full-length cDNAs of 32 of the annotated *CsWRKY *genes (Table 1). Consequently, annotation errors of 17 putative WRKY genes could be corrected (data not shown). All CDSs of 32 *CsWRKY *genes have been submitted to GenBank and their accession numbers in GenBank were showed on Table 1.

**Table 1:** WRKY genes in cucumber

Gene	Annotation ID	GenBank accession	Predicted ORF length	Predicted gene length*	EST hits	Expressed**	Obtained CDS sequence***
*CsWRKY1*	*Csa005379*		1773	3659		-	
*CsWRKY2*	*Csa004516*		1731	2527	4	+	
*CsWRKY3*	*Csa003764*		1839	3302		-	
*CsWRKY4*	*Csa016371*	GU984009	1521	3200	6	+	+
*CsWRKY5*	*Csa015868*	GU984010	828	1150	2	+	+
*CsWRKY6*	*Csa017345*	GU984011	858	1027		+	+
*CsWRKY7*	*Csa001650*		804	2800	1	+	
*CsWRKY8*	*Csa006570*		2184	10512	1	+	
*CsWRKY9*	*Csa026380*	GU984012	1047	1704		+	+
*CsWRKY10#*	*Csa014665*						
*CsWRKY11*	*Csa005866*		768	1648		-	
*CsWRKY12*	*Csa005867*	GU984014	540	953	1	+	+
*CsWRKY13*	*Csa005948*		399	630		+	
*CsWRKY14*	*Csa001212*	GU984015	882	1364	1	+	+
*CsWRKY15*	*Csa018420*	GU984016	681	758	2	+	+
*CsWRKY16##*	*Csa018419*		1506	2683			
*CsWRKY17*	*Csa020112*	GU984017	1581	6663	1	+	+
*CsWRKY18*	*Csa000336*	GU984018	1005	1202	1	+	+
*CsWRKY19*	*Csa008740*	GU984019	1239	2839	1	+	+
*CsWRKY20*	*Csa019944*		849	1123		+	
*CsWRKY21*	*Csa004863*	GU984020	948	1321	2	+	+
*CsWRKY22*	*Csa004896*	GU984021	843	962	2	+	+
*CsWRKY23*	*Csa004828*	GU984022	1431	2653	1	+	+
*CsWRKY24*	*Csa004742*	GU984023	1473	2219	1	+	+
*CsWRKY25*	*Csa002274*	GU984024	939	1614	1	+	+
*CsWRKY26*	*Csa002896*	GU984025	645	1198		+	+
*CsWRKY27*	*Csa002813*		873	1123		+	
*CsWRKY28*	*Csa016219*		315	1475		+	
*CsWRKY29*	*Csa016218*		810	1328		-	
*CsWRKY30*	*Csa010443*		840	2017		-	
*CsWRKY31*	*Csa020355*		1068	1737		+	
*CsWRKY32*	*Csa014848*	GU984026	975	2909	1	+	+
*CsWRKY33*	*Csa009473*	GU984027	1152	1559	1	+	+
*CsWRKY34*	*Csa016087*	GU984028	822	2410		+	+
*CsWRKY35*	*Csa016061*		954	5996		+	
*CsWRKY36*	*Csa015442*		918	1432		+	
*CsWRKY37*	*Csa009672*	GU984029	1521	4068	2	+	+
*CsWRKY38*	*Csa019857*	GU984030	732	3117		+	+
*CsWRKY39*	*Csa019858*		453	592		+	
*CsWRKY40*	*Csa019119*		522	522		+	
*CsWRKY41*	*Csa013101*		510	3539		+	
*CsWRKY42*	*Csa013154*		618	2623		+	
*CsWRKY43*	*Csa010294*	GU984031	546	2318	1	+	+
*CsWRKY44*	*Csa010089*		432	2005		+	
*CsWRKY45*	*Csa010221*		885	1063		-	
*CsWRKY46*	*Csa000701*	GU984032	786	1754	3	+	+
*CsWRKY47*	*Csa003388*	GU984033	897	2148	1	+	+
*CsWRKY48*	*Csa013553*		1449	2980		-	
*CsWRKY49*	*Csa013650*	GU984034	1302	1983	1	+	+
*CsWRKY50*	*Csa007193*	GU984035	876	1554	1	+	+
*CsWRKY51*	*Csa016725*	GU984036	1056	1726	1	+	+
*CsWRKY52*	*Csa001863*	GU984037	729	2911		+	+
*CsWRKY53*	*Csa018657*	GU984038	741	2095	1	+	+
*CsWRKY54*	*Csa018622*	GU984039	240	1886		+	+
*CsWRKY55*	*Csa018069*	GU984040	807	2807	1	+	+
*CsWRKY56*	*Csa018094*	GU984041	498	2565		+	+
*CsWRKY57*	*Csa022995*		972	1454		+	

Note:

* Include intron length;

** Expression of WRKY genes was detected in a variety of cucumber tissues by RT-PCR. +: expressed WRKY genes, -: no signal was detected;

*** The CDS of WRKY genes obtained by RT-PCR; +: obtained.

**# **Annotated *CsWRKY9 *and *CsWRKY10 *were actually one gene.

**## ***CsWRKY15 *and *CsWRKY16 *were two domains of one WRKY gene.

### Multiple sequence alignment, structure and phylogenetic analysis

**Figure 2:**
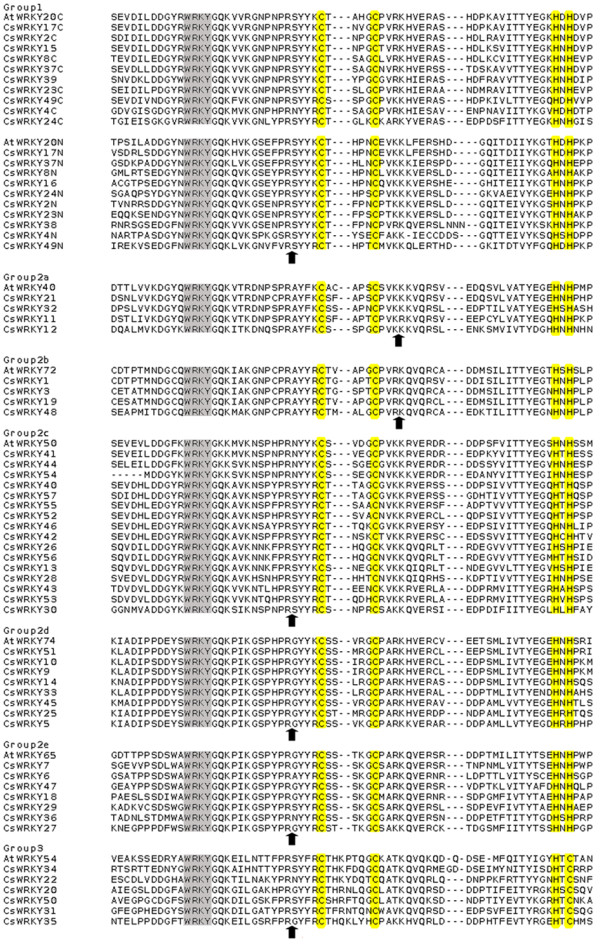
**Alignment of multiple *CsWRKY *and selected *AtWRKY *domain amino acid sequences**. Alignment was performed using Clustal W. The suffix 'N' or 'C' indicates the N-terminal WRKY domain or the C-terminal WRKY domain, respectively, of a specific WRKY protein. The amino acids forming the zinc-finger motif are highlighted in yellow. The conserved WRKY amino acid signature is highlighted in grey, and gaps are marked with dashes. The position of a conserved intron is indicated by an arrowhead.

The phylogenetic relationship of the *CsWRKY *proteins was examined by multiple sequence alignment of their WRKY domains, which span approx 60 amino acids (Figure 2). A comparison with the WRKY domains of several different *AtWRKY *proteins resulted in a better separation of the different groups and subgroups. For each of the groups or subgroups, 1, 2a to 2e and 3, one representative was chosen randomly. These were: *AtWRKY20, 40, 72, 50, 74, 65 *and *54*. As shown in Figure 2, the sequences in the WRKY domain were highly conserved.

**Figure 3:**
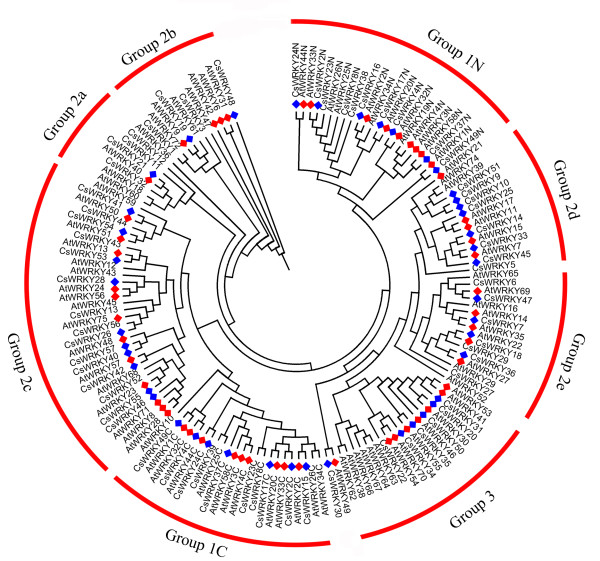
**Unrooted phylogenetic tree representing relationships among WRKY domains of cucumber and *Arabidopsis***. The amino acid sequences of the WRKY domain of all *CsWRKY *and *AtWRKY *proteins were aligned with Clustal W and the phylogenetic tree was constructed using the neighbor-joining method in MEGA 4.0. Group 1 proteins with the suffix 'N' or 'C' indicates the N-terminal WRKY domains or the C-terminal WRKY domains. The red arcs indicate different groups (or subgroups) of WRKY domains. Diamonds represent orthologs from cucumber (blue) and *Arabidopsis *(red).

Sequence comparisons, phylogenetic and structural analyses showed that the WRKY domains could be classified into three large groups corresponding to groups 1, 2 and 3 in *Arabidopsis *as shown by Eulgem *et al.*, 2000 (Figure 3). It is worth noting that group 1 contained 12 *CsWRKY *proteins, eight of which contained two WRKY domains. However, the other four (*CsWRKY15, CsWRKY16, CsWRKY38 *and *CsWRKY39*) contained only one WRKY domain but clustered with CTWD (C-terminal WRKY domains) and NTWD (N-terminal WRKY domains) respectively. Our study further showed that *CsWRKY15 *and *CsWRKY16 *were actually two domains of one WRKY protein, while *CsWRKY38 *and *CsWRKY39 *were two independent WRKY proteins. Domain acquisition and domain loss events appear to have shaped the WRKY family [41,42]. Thus, *CsWRKY38 *and *CsWRKY39 *may have arisen from a two-domain WRKY protein that lost one of its WRKY domains during evolution. The structure and phylogenetic tree of the *CsWRKY *domain clearly indicated that group 2 proteins can be divided into five distinct subgroups (2a-e). Compared with the group 3 proteins in *Arabidopsis *(14 members), there are only 6 *CsWRKY *proteins in group 3. Whereas genome duplication events have resulted in the expansion of the WRKY genes in *Arabidopsis *and rice [17], it appears that these events have not occurred in the cucumber WRKY family. Although Huang *et al. *[40] reported that the cucumber genome shows no evidence of recent whole-genome duplication and tandem duplication. We used the method of Schauser *et al. *[43] to search for small duplication blocks in *CsWRKY *family, but none were found. In addition, a rooted phylogenetic tree of WRKY domains was also constructed to identify putative orthologs in *Arabidopsis *and cucumber (additional file 1). All orthologs are listed in additional file 2.

**Figure 4:**
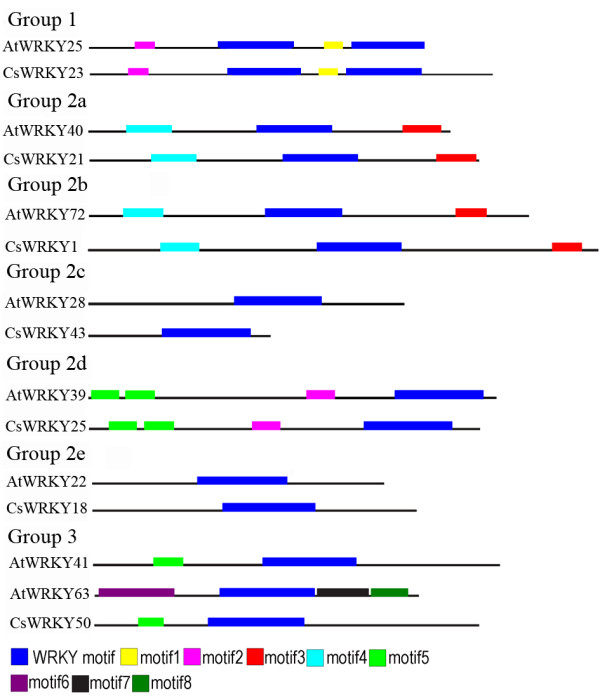
**Schematic diagram of amino acid motifs of *CsWRKY *and *AtWRKY *proteins from different groups (or subgroups)**. Motif analysis was performed using Meme 4.0 software as described in the Methods. The selected WRKY proteins are listed on the left. The black solid line represents the corresponding WRKY protein and its length. The different-colored boxes represent different motifs and their position in each WRKY sequence. A detailed motif introduction for all *CsWRKY *proteins is shown in additional file 4.

Analysis of the structure of C*sWRKY *genes showed that all WRKY genes except *CsWRKY40 *had at least one intron insert. Two major types of intron splicing were found in the conserved WRKY domains of *CsWRKY *genes (Figure 2), which are similar to WRKY domains in *AtWRKY *genes. However, the length of the conserved introns was 2.8 times greater in cucumber (~686 bp) than in *Arabidopsi*s (~241 bp). Coincidentally, this rate was very similar to the size difference (2.9 times) between the genome of cucumber (376 Mb) and *Arabidopsis *(125 Mb). The conserved motifs of WRKY family proteins in cucumber and *Arabidopsis *were investigated using Meme version 4.4 as described in the Methods (additional file 3), and a schematic overview of the identified motifs is given in additional file 4. As displayed schematically in Figure 4, except for the members of group 2c and group 2e, one or more conservative motifs outside of the WRKY domain motif can be detected in a WRKY protein. The *CsWRKY *and *AtWRKY *proteins from the groups 1 and 2, always share the same conserved motifs. In contrast, the members of group 3 *AtWRKY *(*AtWRKY63, AtWRKY64, AtWRKY66 and AtWRKY67*) show an *Arabidopsis-*specific conserved motifs (motifs 6, 7 and 8; additional file 3), but other members of group 3 share the same conserved motifs with other *CsWRKY *proteins.

### Expression profile of *CsWRKY *genes under normal growth conditions and under various abiotic stress conditions

**Figure 5:**
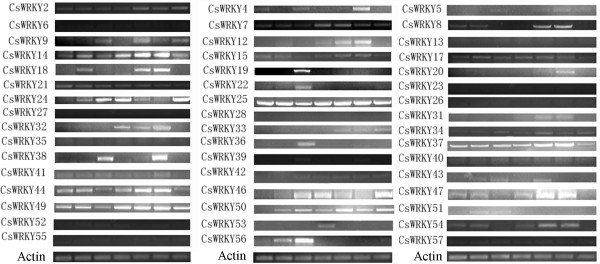
**Expression profiles of cucumber WRKY genes in various tissues as determined by RT-PCR analyses**. Seven amplified bands from left to right for each WRKY gene represent amplified products from cotyledons, leaves, roots, stems, female flowers, male flowers and fruits.

We analyzed the expression of all *CsWRKY *genes under normal growth conditions in seven different tissues: cotyledons, leaves, roots, stems, female flowers, male flowers and fruits. Not all of the predicted genes were expressed in plants grown under normal growth conditions. Among 55 predicted genes, 48 genes (87%) were expressed in at least one of the seven tissues (Figure 5). The other seven genes did not show any detectable expression as tested by RT-PCR in the above tissues, but they may be expressed in other tissues, e.g., seeds. Also, some of the *CsWRKY *genes may be pseudogenes. The following ten genes were expressed in all tested tissues with relatively higher expression intensities: *CsWRKY2, CsWRKY7, CsWRKY14, CsWRKY17, CsWRKY25, CsWRKY37, CsWRKY41, CsWRKY44, CsWRKY49 *and *CsWRKY57*. Five WRKY genes (*CsWRKY5, CsWRKY13, CsWRKY23, CsWRKY28 *and *CsWRKY55*) were expressed at relatively low levels in all the tested tissues.

**Figure 6:**
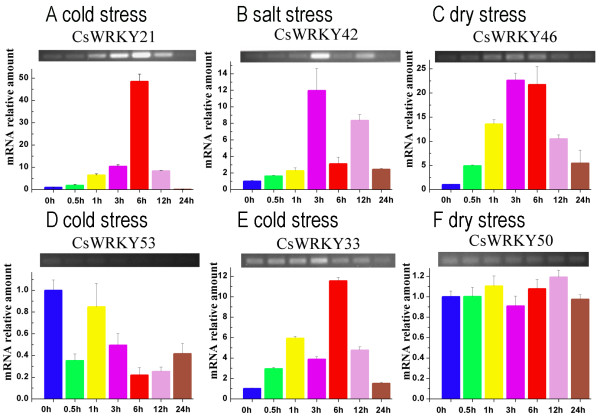
**Expression patterns of six selected WRKY genes under abiotic stresses**. In A-F, the top panel shows the RT-PCR result and the bottom panel shows the corresponding real-time PCR result. For real-time PCR, the relative amount of mRNA (y-axis) was calculated by according to the description in Methods. The cucumber *β-actin *gene was used as an internal control to normalize the data. The 0, 0.5, 1, 3, 6, 12, and 24 (x-axis) indicate the treatment time (hour) under corresponding abiotic stresses. The error bars were calculated based on three replicates. A-C, significant up-regulated expression of WRKY genes can be detected under abiotic stresses. D, significant down-regulated expression of *CsWRKY53 *can be detected under cold treatment. E, the expression difference detected by real-time PCR was more significant than that detected by RT-PCR. F, no significant expression difference can be detected in group 3 WRKY gene *CsWRKY50 *under abiotic stress. Statistical significance was obtained by using Student's t-test.

We used RT-PCR analyses to examine the expression of *CsWRKY *genes in response to three different abiotic stresses: cold, drought and salinity. Of the 48 expressed *CsWRKY *genes, 23 showed differential expressions in response to at least one stress, whereas the other 25 did not (Table 2). It should be noted that none of the stress-inducible *CsWRKY *genes belongs to group 3. We conducted real-time PCR analyses to confirm and quantify the expression levels of the 23 stress-inducible WRKY genes in response to abiotic stresses. As shown in Figure 6, RT-PCR and real-time PCR generally gave the same results for the expression profiles and abundance of transcripts. However, in rare instances, the difference in expression detected by real-time PCR was more significant than that detected by RT-PCR (Figure 5E). As shown in Table 2, the results of real-time PCR showed that most of the stress-responsive genes were upregulated in response to abiotic stress (Figure 6A, B, C), and only three genes were downregulated (Figure 6D). As determined by real-time PCR analysis, there were no differences in the expressions of six group 3 *CsWRKY *genes in response to abiotic stress (Figure 6F).

**Table 2:** *CsWRKY *gene expression patterns under abiotic stress as determined by RT-PCR and real-time PCR.

Gene	Cold	Salt	Dry	Gene	Cold	Salt	Dry
*CsWRKY2*	+	+	+	*CsWRKY32*	nc	nc	nc
*CsWRKY4*	+	nc	nc	*CsWRKY33*	+	nc	nc
*CsWRKY5*	nc	nc	nc	*CsWRKY34*	nc	nc	nc
*CsWRKY6*	nc	nc	nc	*CsWRKY35*	nc	nc	nc
*CsWRKY7*	nc	nc	nc	*CsWRKY36*	+	nc	nc
*CsWRKY8*	nc	nc	nc	*CsWRKY37*	nc	nc	nc
*CsWRKY9*	nc	nc	nc	*CsWRKY38*	nc	nc	nc
*CsWRKY12*	nc	nc	nc	*CsWRKY39*	nc	+	+
*CsWRKY13*	nc	nc	nc	*CsWRKY40*	++	++	++
*CsWRKY14*	nc	+	+	*CsWRKY41*	nc	+	nc
*CsWRKY15*	nc	nc	nc	*CsWRKY42*	nc	+	nc
*CsWRKY17*	nc	nc	nc	*CsWRKY43*	nc	+	+
*CsWRKY18*	++	+	++	*CsWRKY44*	nc	+	+
*CsWRKY19*	nc	nc	nc	*CsWRKY46*	+	++	+
*CsWRKY20*	nc	nc	nc	*CsWRKY47*	nc	nc	nc
*CsWRKY21*	++	++	++	*CsWRKY49*	nc	nc	nc
*CsWRKY22*	nc	nc	nc	*CsWRKY50*	nc	nc	nc
*CsWRKY23*	+	-	nc	*CsWRKY51*	nc	nc	nc
*CsWRKY24*	nc	nc	nc	*CsWRKY52*	nc	+	+
*CsWRKY25*	++	nc	nc	*CsWRKY53*	-	nc	+
*CsWRKY26*	nc	nc	nc	*CsWRKY54*	nc	+	+
*CsWRKY27*	nc	nc	nc	*CsWRKY55*	-	nc	++
*CsWRKY28*	-	nc	nc	*CsWRKY56*	nc	+	+
*CsWRKY31*	nc	nc	nc	*CsWRKY57*	++	nc	+

Cucumber seedlings were subjected to salt, drought and cold treatments for 0, 0.5,1, 3, 6 12 and 24 h.

Note:

nc, no significant change in gene expression; +, moderate induction of gene expression; ++, strong induction of gene expression; -, reduction of gene expression.

Student's t-test was used to obtain the statistical significance of the difference between treated samples and untreated samples (0 h treatment under abiotic stress). If P-values < 0.01, we considered the WRKY gene as an induced gene.

### Comparison of abiotic stress-inducible orthologs between cucumber and *Arabidopsis*

**Figure 7:**
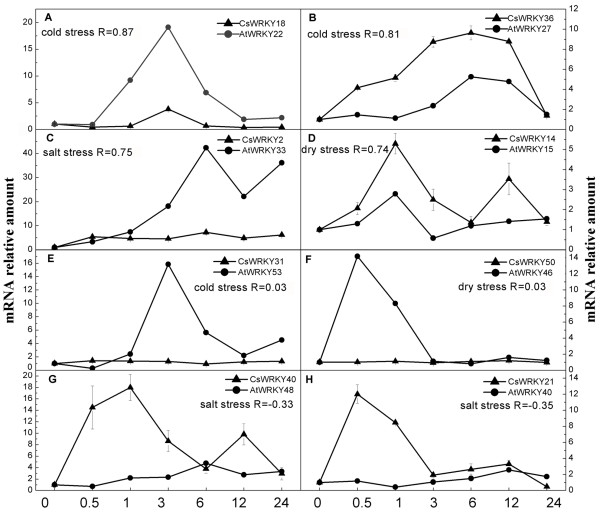
**Pairwise comparisons of the expression profiles of putative orthologous cucumber and *Arabidopsis *WRKY genes under abiotic stresses**. The relative expression of CsWRKY genes was obtained by real-time RT-PCR (indicated by triangles). Data are the means of three replicates with standard errors represented by bars. The CsWRKY expression data were compared with the mean-normalized expression data for their putative orthologous AtWRKY genes from a publicly available Arabidopsis microarray data set (indicated by circles) according to the description in Methods. The relative amount of mRNA (y-axis) was the ratio of treated to untreated sample. The treatment time (h) under the particular abiotic stress is presented on the x-axis. R indicates the correlation coefficient for expression between orthologs under the corresponding abiotic stresses. A distinct positive correlation was detected in most orthologs (A-D), but no obvious correlation was detected in group 3 orthologs (E-F). A negative correlation was detected in a small number of orthologs (G-H).

We compared the expressions of *CsWRKY *genes with those of their possible orthologs in *Arabidopsis *under abiotic treatment. As shown in additional file 5, except for group 3 WRKY genes, *Arabidopsis *WRKY genes whose orthologus *CsWRKY *genes were not induced by abiotic treatments were also not stresses-inducible. In addition, most of orthologous *AtWRKY *genes of stress-inducible CsWRKY genes also responded to at least one stress-type treatment. These findings imply a possible correlation between the expression profiles of these orthologs in *Arabidopsis *and cucumber in response to abiotic stresses. Among the *CsWRKY *genes whose expressions changed in response to abiotic stress, there were 13 for which stresses-inducible orthologs existed in *Arabidopsis *(additional file 5). To investigate whether the expressions of these orthologs were correlated between the two species, we compared the expressions of these 13 pairs of orthologs under various stresses as described in the Methods section. This analysis generated a total of 22 sets of data (one pairs of orthologs may be induced by more than one abiotic stresses). As shown in Table 3, the correlation coefficients of 12 sets of data, more than half of the 22 sets of data, were greater than 0.5, indicating a positive correlation between the orthologous pairs under abiotic stresses (Figure 7A-D). The expression profiles of only two sets of data were negatively correlated (Figure 7G-H). Finally, the average correlation coefficients of 22 datasets for all the putative orthologous WRKY genes was 0.40 and differed significantly (p < 0.01) from the average expression correlation of a control dataset composed of randomly chosen gene pairs (0.04) (Table 3). In contrast, when the correlation coefficients of group 3 *CsWRKY *and *AtWRKY *orthologs were calculated, there was no clear positive or negative correlation (Figure 7E-F). Our results indicated that there is a correlative expression profile between stress-inducible *CsWRKY *genes and their putative *AtWRKY *orthologs, except for the group 3 WRKY genes. This finding suggests that the expression of group 3 WRKY orthologs differ between cucumber and *Arabidopsis*. All expression data used to calculate correlations are shown in additional file 6.

**Table 3:** Pearson correlation coefficients for expression profiles of orthologs*

CsWRKY	AtWRKY	Stresses	Correlation coefficient
*CsWRKY18*	*AtWRKY22*	cold	0.87
*CsWRKY36*	*AtWRKY27*	cold	0.81
*CsWRKY33*	*AtWRKY7*	cold	0.77
*CsWRKY2*	*AtWRKY33*	salt	0.75
*CsWRKY14*	*AtWRKY15*	dry	0.74
*CsWRKY42*	*AtWRKY57*	salt	0.70
*CsWRKY21*	*AtWRKY40*	cold	0.67
*CsWRKY55*	*AtWRKY23*	cold	0.66
*CsWRKY2*	*AtWRKY33*	dry	0.62
*CsWRKY57*	*AtWRKY48*	dry	0.61
*CsWRKY25*	*AtWRKY11*	cold	0.60
*CsWRKY4*	*AtWRKY32*	cold	0.52
*CsWRKY57*	*AtWRKY48*	cold	0.45
*CsWRKY40*	*AtWRKY48*	dry	0.40
*CsWRKY21*	*AtWRKY40*	dry	0.34
*CsWRKY46*	*AtWRKY28*	dry	0.14
*CsWRKY40*	*AtWRKY48*	cold	0.01
*CsWRKY2*	*AtWRKY33*	cold	-0.08
*CsWRKY25*	*AtWRKY17*	cold	-0.09
*CsWRKY18*	*AtWRKY22*	dry	-0.11
*CsWRKY40*	*AtWRKY48*	salt	-0.33
*CsWRKY21*	*AtWRKY40*	salt	-0.35
Average correlation stress-induced othologous WRKY gene pairs			0.40
Average correlation random genes**			0.04

*Available expression data on *AtWRKY *genes from microarray analysis and that of *CsWRKY *genes generated by real-time PCR analysis were used to calculate the Pearson correlation coefficient for the expression of orthologous WRKY genes under various abiotic stresses (after 0, 0.5, 1, 3, 6, 12, and 24 h treatment)(as showed in Figure 7)as described in the Methods.

**a randomly chosen abiotic stress induced cucumber WRKY gene and a randomly chosen abiotic stress induced *AtWRKY *gene composed of a random gene pair. This process was repeated a 100 times and produced 100 random WRKY gene pairs. The expression correlation of each of 100 random WRKY gene pair was calculated as described in the Methods

### Evolutionary analysis of group 3 WRKY genes in *Arabidopsis *and cucumber

**Figure 8:**
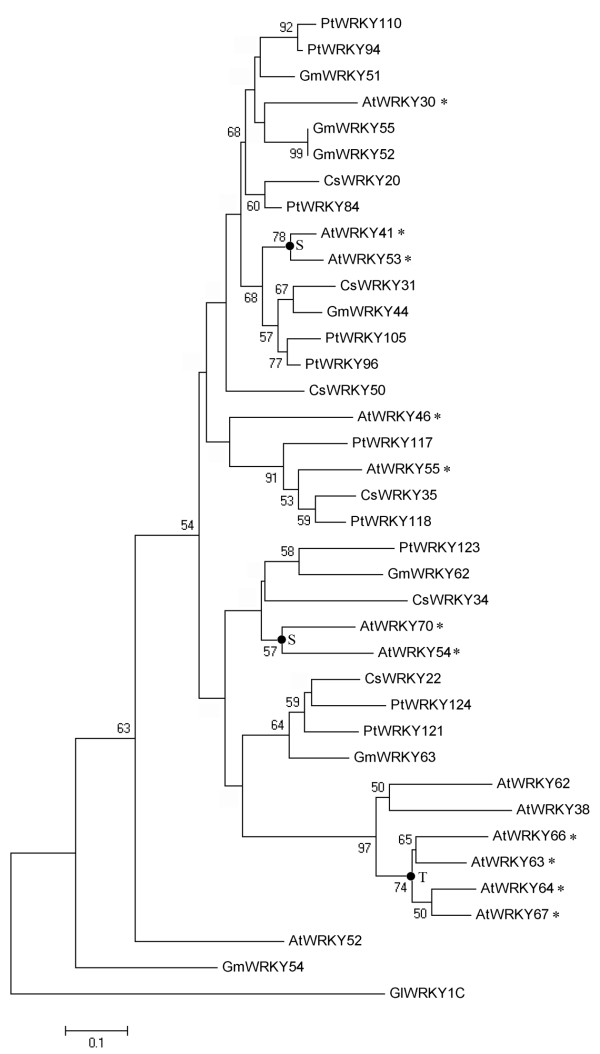
**Phylogram of group 3 WRKY domains from *Arabidopsis *(*AtWRKY*), cucumber (*CsWRKY*), poplar (*PtWRKY*) and soybean (*GmWRKY*)**. The phylogenetic tree was constructed using the neighbor-joining method as implemented in PHYLIP 3.2. Numbers on internal nodes are the percentage bootstrap support values (1000 re-sampling); only values exceeding 50% are shown. The most primitive *Giardia lamblia *WRKY C-terminal domain (*GlWRKY*1C) was used as an outgroup. The letters T and S indicate nodes where tandem duplication and recent segmental duplication events have occurred, respectively. * indicates the *AtWRKY *associated with the gene duplication events.

The group 3 WRKY genes seem to have greatly expanded in angiosperms after the divergence of the monocots and dicots (160 Mya) [44]. Here, we further investigated the duplication and diversification of group 3 WRKY genes after divergence of the eurosids I group (which include cucumber, soybean, and poplar) and the eurosids II group (which include *Arabidopsis*) (110 Mya). A phylogenetic tree of WRKY proteins encoded by group 3 WRKY genes of *Arabidopsis *(14), cucumber (6), poplar (10), and soybean (7) was constructed using the most primitive WRKY domain of *Giardia lamblia *as an outgroup. This analysis showed that many members of the group 3 AtWRKY proteins clustered together and displayed the close phylogenetic relationship (Figure 8), indicating that they arose after the divergence of the eurosids I and II. Two types of gene duplication events, tandem duplication and segmental duplication, were the main factors in the expansion of group 3 *AtWRKY *genes. The results of this phylogenetic analysis indicated that no gene duplication events have occurred in *CsWRKY *gene evolution because of no paralogs of cucumber can be detected. Hence, the different evolutionary patterns of group 3 WRKY in cucumber and *Arabidopsis *occurred after their divergence.

**Figure 9:**
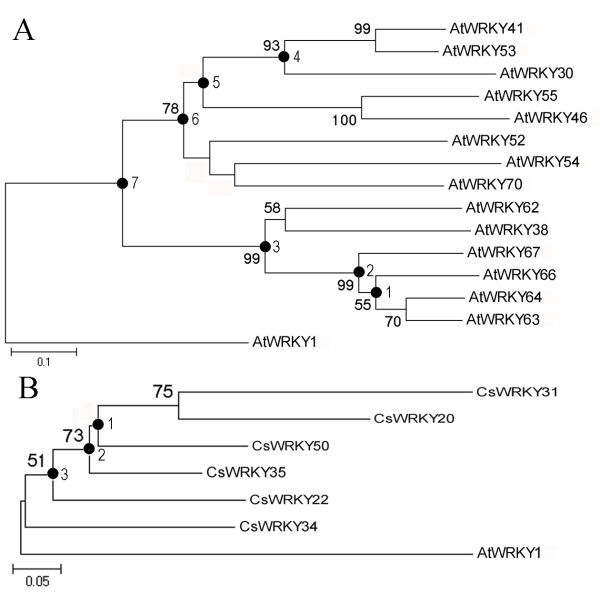
**Phylogram of group 3 WRKY genes of *Arabidopsis *and cucumber**. The phylograms were constructed using the neighbor-joining method as implemented in PHYLIP 3.2. Numbers on the left of each internal node represent bootstrap support values (1000 re-sampling); only values exceeding 50% are shown. Numbers on the right of each node represent the nodes that were used for positive selection analysis. *Arabidopsis AtWRKY1 *was used as an outgroup. The trees represent phylogenetic relationships among (A) *AtWRKY *proteins and (B) *CsWRKY *proteins.

To determine whether selection pressure had affected group 3 WRKY genes, we estimated the ω (dn/ds) values for all branches of group 3 WRKY genes in *Arabidopsis *and cucumber (Figure 9 and Table 4). In *Arabidopsis*, the ML estimate of dN/dS values for all nodes under model M0 were < 1, with a mean value of 0.276 (Table 4), indicating that group 3 *AtWRKY *genes have been under purifying selection, which was the predominant force acting on the evolution of the group 3 *AtWRKY *genes. However, the log likelihood differences between model M3 and model M0 were statistically significant for all nodes tested, suggesting that selective pressure varied among branches and some genes might have been under positive selection. We further used model M7 and M8 of PAML to address whether positive selection has played a role in the evolution of group 3 *AtWRKY *genes. Of the eight nodes analyzed, log-likelihood values were significantly higher under the M8 model than under the M7 model for five nodes (nodes 1, 2, 3, 4 and 5), which indicates that positive selection has contributed to the evolution of group 3 *AtWRKY *genes. Interestingly, the terminal nodes with clusters of duplicated *AtWRKY *genes were all under positive position selection, suggesting a correlation between duplication of genes and positive selection. Furthermore, we identified the positively selected sites under model M8 using the Bayesian method. Several positive selection sites were detected in above five nodes but only one positive selection site could be detected in the region of WRKY domains. Thus, it appears that because of the high degree of conservation in WRKY domains of the WRKY genes, the positive selection contributed mostly to the regions outside of the WRKY domains. In cucumber, although the log likelihood differences between model M3 and model M0 suggest that selective pressure varied among branches, there was no detectable positive selection in any of the nodes. Assuming that there were no duplication events in *CsWRKY *genes and that positive selection is associated with duplication of WRKY genes as we described here, the extensive positive selection events were probably followed by the group 3 *WRKY *gene duplication events. This positive selection might be the main evolutionary force for group 3 *AtWRKY *genes. Due to the absence of duplicated genes and positive selection in cucumber, the functions of group 3 *CsWRKY *genes might be more conservative than those of *AtWRKY *genes.

**Table 4:** Likelihood ratio test results of group 3 ***AtWRKY ***and ***CsWRKY***.

Group 3 *AtWRKY *					
**Node^a^**	**dN/dS M0^b^**	**2ΔlnL M3 vs. M0**	**2ΔlnL M8 vs. M7**	**M8 estimates^c^**	**No. of positive selection sites^d^**
1	0.5712	170.69**	17.76**	ω = 2.78 β(p = 0.76 q = 1.17)	7
2	0.5689	36.21**	6.92*	ω = 4.68 β(p = 0.39 q = 0.34)	10
3	0.3248	141.78**	8.37*	ω = 32.95 β(p = 0.37 q = 0.26)	5
4	0.6485	54.62**	9.97**	ω = 77.65 β(p = 0.66 q = 1.05)	11
5	0.2682	169.06**	10.66**	ω = 3.32 β(p = 0.72 q = 0.78)	9

Group 3 *CsWRKY *					

Node	dN/dS M0	2ΔlnL M3 vs. M0	2ΔlnL M8 vs. M7	M8 estimates	No. of positive selection sites
1	0.3331	37.31**	1.40e-05	ω = 4.28 β(p = 0.85 q = 1.39)	0
2	0.3623	83.01**	8.80e-05	ω = 1.00 β(p = 0.72 q = 1.143)	0
3	0.3081	186.07**	2.99e-05	ω = 24.88 β(p = 0.60 q = 0.55)	0

Note: * p < 0.05 and ** p < 0.01 (χ2 test)

a Node number from the phylogenetic tree

b dN/dS is the average ratio over sites under a codon model with one ratio

c ω was estimated under model M8; p and q are the parameters of the beta distribution

d The number of amino acid sites estimated to have undergone positive selection under M8

## Discussion

### Whether the *CsWRKY *genes were underrepresented in this study?

The WRKY gene family has 72 members in *Arabidopsis *[1] and 109 members in rice [17]. In this study, we identified a total of 55 *CsWRKY *genes. Compared with *Arabidopsis *(genome size 125 Mb) and rice (genome size 480 Mb), in cucumber (genome size 367 Mb), the size of the WRKY family is small. We further compared the number of WRKY genes in different subgroup among *Arabidopsis*, rice, grape and cucumber (Table 5). As showed in table 5, the key difference is that the number of group 3 *CsWRKY *genes (6) was much lesser than those of *Arabidopsis *(14) and rice (36). A problem has arisen. Whether *CsWRKY *genes, especially group 3 *CsWRKY *genes, are underrepresented or not in our study?

**Table 5:** The number of WRKY in cucumber, *Arabidopsis*, grape and rice

	Group1	Group2a	Group2b	Group2c	Group2d	Group2e	Group3
CsWRKY	10	4	4	16	8	7	6
AtWRKY	13	4	7	18	7	9	14
VvWRKY*	12	4	7	14	6	7	5
OsWRKY	15	4	8	15	7	11	36

Note: * the WRKY proteins of grape (*Vitis vinifera*)

Complete and accurate annotation of genes is an essential starting point for further evolution and function study in gene family. We identified a total of 55 *CsWRKY *genes from 26682 cucumber annotated genes in cucumber genome. In addition, a total of 357882 cucumber EST sequences download from Cucumber Genome DataBase and NCBI were used to test whether there are new WRKY proteins encoded by these EST sequences that were ignored in our annotation for *CsWRKY *proteins. The amino acid sequences of the open reading frame (ORF) of the EST were subjected to HMM program search. The results were screened manually for false positives at E values above 10^100^. Even with this weak criterion, we failed to find any new WRKY proteins in cucumber genome, which indicate that the annotation for cucumber WRKY genes is complete. We further used experimental methods to test the accuracy of annotation for *CsWRKY *genes. According to the annotated WRKY genes sequence, we detected the expression of 48 *CsWRKY *genes (87%), indicating that the accuracy of annotation for *CsWRKY *genes is high. Moreover, we cloned and sequenced full-length cDNAs of 32 of the annotated *CsWRKY *genes (Table 1), and some annotation errors were corrected. For example, we found that predicted *CsWRKY*15 and *CsWRKY16 *were actually two domains of one WRKY protein. Through this process, the integrity and accuracy of annotated *CsWRKY *genes were improved and were high enough to use in our further study. Therefore, we believed that *CsWRKY *genes would not be underrepresented in our study.

### The quickly expansion of group 3 WRKY genes is associated with the recent duplication events

Many angiosperms underwent whole genome duplication events (γ, β, α). The γ event appears to pre-data monocots-dicots divergence. The β event pre-dated *Arabidopsis *divergence from the other dicots, but post-dated divergence from the monocots about 170-235 Myr ago. The α duplication event (recent duplication events) pre-dated *Arabidopsis *divergence from Brassica about 14.5-20.4 million years (Myr) ago [45]. The recent gene duplication events are most important in the quickly expansion and evolution of gene families [46]. Therefore, in our manuscript, we only analyze the influence of recent duplication events to *CsWRKY *genes.

Both *Arabidopsis *and rice genome underwent the recent duplication events, which lead to the large-scale expansion of gene family in their genome [46,47]. Zhang et al. report that group 3 WRKY domains appear to have been duplicated independently after the divergence of monocots and dicots (160 Mya) [44]. In this study, we further study the duplication of group 3 WRKY genes after divergence of the eurosids I group and the eurosids II group (110 Mya). As showed in Figure 7, the close paralogs WRKY genes of *Arabidopsis*, poplar and soybean each clustered together respectively, indicating that the expansion of the group 3 WRKY gene family may have occurred after the divergence of the eurosids I and eurosids II (110 Mya), and should be related to the most recent genome duplication events(24-40 Mya). Moreover, our result indicated that one of important factor in the expansion of group 3 *AtWRKY *was the occurrence of tandem duplication events. Four tandem duplication genes were clustered together in phylogenetic trees, indicating that the tandem duplication occurred after the divergence of the eurosids I and eurosids II and also related with recent duplication events. Interestingly, tandem duplication was an important recent gene duplication pattern in *Arabidopsis *genome [46], but in *AtWRKY *gene family there were only four *AtWRKY *genes from tandem duplication blocks and all of them belonged to group 3 *AtWRKY *genes. From these, we can see that the group 3 *AtWRKY *genes expanded quickly in *Arabidopsis *genome by two duplication patterns: recent segmental duplication and recent tandem duplication, which indicate that group 3 WRKY genes may play important roles in the adaptability of angiosperms.

As far as cucumber concerned, although Huang et al., reported that the cucumber genome was absence of recent whole-genome duplication events and tandem duplication [40]. The method of Schauser [43] was still used to detect whether recent small duplication blocks occur in *CsWRKY *family. We found no *CsWRKY *genes locus on any recent duplication blocks (additional file 2). In addition, from the Figure 1, we can see that there are no tandemly arrayed WRKY genes on the same chromosomal location, which indicate the absence of recent tandem duplication event in *CsWRKY *genes. Therefore, compared with *Arabidopsis *and rice, the size of group 3 *CsWRKY *proteins is small, which can be attributed to the absence of recent duplication events in cucumber genome. To prove this hypothesis, we search the grape WRKY proteins (*VvWRKY*) in grape genome. The grape genome, like cucumber, has not undergone recent duplication events [48]. As showed by table 5, there are only five group 3 *VvWRKY *(*GSVIVT01028718001*, *GSVIVT01019511001*, *GSVIVT01027069001*, *GSVIVT01032662001 *and *GSVIVT01032661001*) can be detected in grape genome. Therefore, on the base of the above discussion, we believe that compared with *Arabidopsis *and rice, the small size of group 3 *CsWRKY *can be attribute to the absence of recent duplication events in cucumber genome rather than the underrepresentation of group 3 *CsWRKY *in our study.

### *CsWRKY *proteins play important roles in various biological processes

The reported WRKY gene (SE71, ID: AAC37515.1) of cucumber shares 93% similarity with the *CsWRKY37 *reported here. The expression of SE71 increases in cotyledons as they expand and become photosynthetic, suggesting an involvement of SE71 in the development of cotyledons and cucumber photosynthesis [7]. Our RT-PCR results showed that *CsWRKY37 *was expressed in all seven cucumber tissues at relatively high levels, which indicates that *CsWRKY37 *could play a role not only in development of cotyledons and photosynthesis but also in the processes such as flower formation and fruit development. Besides *CsWRKY37*, some other CsWRKY genes also showed relative high expression levels in all seven organs, such as *CsWRKY25 *and *CsWRKY49*. The WRKY genes that are highly expressed in plant organs often play key roles in plant development [18]. The role of WKRY gene in plant development is in transcriptional regulation of expression of target genes that are involved in some physiological pathway [3]. So, we speculated that the highly expressed *CsWRKY *genes reported here may play a regulatory role in cucumber development. However, more research is needed to determine the functions of the *CsWRKY *genes.

Evidence is accumulating that WRKY proteins are involved into response to various abiotic stresses. At least 54 *OsWRKY *genes of rice and 26 *GmWRKY *genes of soybean were found to be differentially expressed under abiotic stresses [18]. In this study, we showed that 23 *CsWRKY *genes exhibited differential expression in response to at least one abiotic stress, indicating that *CsWRKY *genes may play an important role in cucumber responding to abiotic stresses. In fact, previous studies indicated that some of the WRKY proteins are stable and resistant to environmental stresses. Huang et al. reported that a WRKY gene of bittersweet nightshade *(STHP-64) *encoded an anti-freeze protein, which contains a unique 13-mer repeat in the C-terminus, known to be a common feature of animal antifreeze proteins [9]. However, increasing number of studies indicate that WRKY proteins are transcriptional factors that regulate the tolerance of plant to abiotic stresses [38]. As shown in Figure 6, some of the *CsWRKY *genes responded to stresses at an early stage. For example, *CsWRKY18 *peaked at 0.5 h after drought treatment. These results indicated that some *CsWRKY *genes possible may be as a transcriptional factor to regulate the tolerance of cucumber to stresses. To understand the biological functions of WRKY transcriptional factors, the identification of target genes and the regulatory network of WRKY transcriptional factors are necessary. The soybean *GmWRKY54 *expressed in transgenic *Arabidopsis *showed that *GmWRKY54 *can regulate the expression of DREB2A, which contains a W-box motif in the promoter region and is known to act as a transcriptional factor regulated the expression of many drought-inducible genes [15]. Other recent studies have revealed that two co-regulated networks exist in rice regulating the response to various abiotic stresses [49]. These results indicate that the regulatory role of WRKY proteins under abiotic stresses is complex and more work is needed to understand the regulatory mechanisms.

### The functional conservative and divergence of orthologous genes between *Arabidopsis *and cucumber

In comparative genomics, the clustering of orthologous genes highlights the divergence and conservation of gene families among multiple genomes. Two strategies have often been used to identify orthologs or paralogs: phylogeny-based methods and BLAST-based methods [50]. The comparison of results from phylogeny-based methods contains widely orthologous pairs information but may lead to false positives error [51]. Therefore strict criteria must be adopted in phylogeny-based methods. BLAST-based method (Bi-direction best hit) shows a good overall performance but is restricted to 1:1 orthologs which may lead to omit the in-paralogs [51]. In this study, a rooted phylogenetic tree based on WRKY domain of rice, cucumber and *Arabidopsis *was used to arrange possible orthologs of cucumber and *Arabidopsis*. In addition, a standard approach BBH (bidirectional best hit) was also used as reference to arrange possible orthologs. Relatively strict criteria were used to arrange orthologus genes in this study. The nodes of phylogenetic tree which the bootstrap support values (1000 re-sampling) exceed 50% were used to identify possible orthologs pairs. For example, *AtWRKY65 *and *CsWRKY6 *were clustered together in phylogenetic tree, but the bootstrap of their node is no more than 50%. Therefore, *AtWRKY65 *and *CsWRKY6 *were excluded from the orthologous pair, so does *CsWRKY11 *and *AtWRKY18/60*. In addition, the members of group 1 WRKY were considered as possible orthologous pairs unless the same phylogenetic relationship can be detected between their N-domain and C-domain in the phylogenetic tree. For example, *CsWRKY8 *and *AtWRKY25 */*26 *were excluded from orthologous pairs because of the different cluster of their N-domain and C-domain in the phylogenetic tree. Totally, we found 38 orthologus pair between cucumber and *Arabidopsis *(additional file 2).

We further analyze the correlation of orthologous pairs under abiotic stresses. Our results show that correlative expression profiles in stress-inducible orthologous WRKY genes between cucumber and *Arabidopsis*. Mangelsen et al. reported that in homologous organs the average correlation coefficient of the orthologous WRKY genes between monocots and dicots can reach 0.24 [52]. Because researches on the role played by cucumber genes in abiotic stress tolerance are quite limited, our study provide a new starting point for investigating the function of cucumber genes by comparing the orthologous genes between cucumber and *Arabidopsis*. Furthermore, in our study, orthologous WRKY genes with different evolution patterns displayed a low correlation in their expression patterns. Almost half of *CsWRKY *genes in our study responded to at least one abiotic stresses, but none of them belongs to group 3. In contrast, the expression data from microarray of *AtWRKY *genes has revealed that all the gene orthologous to group 3 *CsWRKY *genes response to abiotic stresses in *Arabidopsis*, and interestingly all of them are located in a recent segmentally duplicated region. The recent Segmental duplication occurs most frequently in plants because most plants are diploidized polyploids and retain numerous duplicated chromosomal blocks in their genomes [53]. As discussed earlier in this paper, after the divergence of eurosids I and eurosids II, the group 3 *AtWRKY *genes experienced segmental duplication events. The long-term evolutionary fate of duplication genes will be determined by functions of the duplicated genes. Four types of functional differentiation may follow by gene duplication: pseudogenization, conservation of gene function, subfunctionalization and neofunctionalization [54]. Many duplicated genes may be lost from the genome after the duplication events, and neofunctionalization and subfunctionalization are the major factors for the retention of new genes. In addition, positive selection may play important roles in the neofunctionalization and subfunctionalization of duplication genes. In the case of neofunctionalization of duplicated genes, positive selection accelerates the fixation of advantageous mutations that enhance the activity of the novel function. In the case of subfunctionalization of duplicated genes, each daughter gene will inherit one of functions of ancestral gene, and further substitutions under positive selection can refine the functions [47]. In *Arabidopsis*, the number of group 3 WRKY genes increased significantly due to the duplication events after divergence of the eurosids I and eurosids II, and our results suggested that all duplicated group 3 *AtWRKY *experienced a positive selection after their duplication events. The retention of new members of group 3 *AtWRKY *could be contributed to their neofunctionalization. In rice, high expression divergence could be one of the mechanisms for the retention of duplicated WRKY genes [18]. Due to the lack of gene duplication events in the *CsWRKY *family, the functions of group 3 *CsWRKY *genes are probably more conservative than that of *AtWRKY*. The functions of the group 3 *CsWRKY *genes likely resemble the functions of a common ancestor that existed before the divergence of eurosids I and II. Indeed, the common ancestor may not have been responsive to abiotic stresses, and the stress-responsive ability of the group 3 *AtWRKY *genes could be due to neofunctionalization following gene duplication event(s).

## Conclusions

In this study, we identified a total of 55 cucumber WRKY genes and analyzed the expression profile of 48 *CsWRKY *genes under normal growth conditions and in response to various abiotic stresses. These new WRKY sequences and expression information reported here will be useful for further investigating the function of WRKY genes under various stress conditions. Although the genome sequence of cucumber has been reported, functional studies on cucumber genes are still lag behind. Our results show that correlative expression profiles exist between putative WRKY orthologs of cucumber and *Arabidopsi*s. Hence, comparative genomics approaches could be used to investigate gene function. In addition, compared with group 1 and 2 WRKY genes, the group 3 WRKY genes seem to have arisen more recently in angiosperms, but have expanded rapidly. Our results also indicate that positive selection could have led to the functional divergence of duplicated genes during the expansion of group 3 WRKY genes. Based on all the results presented here, we speculated that the functional divergence of WRKY proteins has played a critical role in the responses of plants to various stresses.

## Methods

### Sequence database searches

*Arabidopsis *WRKY proteins sequences were obtained from TAIR [55]. The rice WRKY proteins sequences were obtained from rice genome annotation project [56]. The WRKY proteins of poplar and soybean were obtained from PFAM database [57]. The GenBank accession numbers of WRKY protein sequences were provided in additional file 7. The WRKY proteins of grape were obtained from http://www.genoscope.cns.fr/externe/Download/Projets/Projet_ML/data/12X/annotation/Vitis_vinifera_peptide.fa.gz.

The cucumber annotated (predicted) genes and proteins were obtained from Cucumber Genome Sequencing Project which we participated in. Now, this annotated data can be downloaded from Cucumber Genome DataBase [58]. We searched WRKY proteins from a total of 26682 predicted cucumber proteins. We used 72 *Arabidopsis *WRKY proteins as query sequences and Blastp searches against the predicted cucumber proteins. The sequences were selected as candidate proteins if their E value satisfied E was ≤-10. Based on the HMMER User's Guide, the Hmmsearch program was then used to predict the WRKY domains (PF03106.7) of all these candidate proteins and the E valve was set to -10. The new WRKY-like sequences confirmed by Hmmsearch in the cucumber genome were in turn used reiteratively to search the cucumber predicted proteins until no new sequences were found. The EST sequences of cucumber were downloaded from NCBI and Cucumber Genome DataBase [58].

### Multiple sequence alignment, gene structure construction and phylogenetic analysis

The 60 amino acid spanning WRKY core domain of all *CsWRKY *proteins and selected *AtWRKY *protein (*AtWRKY20 (At4g26640), 40 (At1g80840), 72 (At5g15130), 50 (At5g26170), 74 (At5g28650)*, *65 (At1g29280) *and *54 (At2g40750)) *was used to create multiple protein sequence alignments using ClustalW [59]. Default settings were applied for the alignment in Figure 2. The gene structure was obtained by the cucumber gene annotation GIFF3 file downloaded from Cucumber Genome DataBase. The neighbor-joining method was used to construct the phylogenetic tree based on amino acid sequence of WRKY domains. Two types of software, MEGA 4.0 and PHYLIP 3.2 were used [60,61]. The MEGA 4.0 analysis was carried out according to the description by Zhang *et al.*, [62] and the PHYLIP 3.2 analysis was carried out according to the description by Zhou *et al.*, [15]. Motif detection was performed with MEME 4.0 software [63]. A rooted phylogenetic tree based on WRKY domain of rice, cucumber and *Arabidopsis *was used to arrange possible orthologs of cucumber and *Arabidopsis*. In addition, a standard approach BBH (bidirectional best hit) was also used as reference to arrange possible orthologs [51,64].

### Microarray based expression analysis and correlation calculation

For the expression analysis of *AtWRKY *genes, publicly available microarray data of the AtGenExpress global stress expression data set [37] were used. The microarray data of cold stress (ME00325), drought stresses (ME00338) and salt stresses (ME00328) were downloaded from Weigel World database [65]. The mean-normalized values of the expression data were used in further analysis. The relative amount of mRNA was calculated by dividing the expression data of the stress treatment by that of the control (0 h treatment).

Available expression data on *AtWRKY *genes from microarray analysis and that of *CsWRKY *genes generated by real time RT-PCR analysis described here were used to calculate the Pearson correlation of the expression of orthologous *WRKY *genes. All expression data (relative amount of mRNA) are composed of seven treatment points (0, 0.5, 1, 3, 6, 12, and 24 h) under corresponding abiotic stresses. For each of orthologous WRKY gene pairs, the correlation of the expression data under their corresponding abiotic stresses was calculated. The following methods were used to test the significance of correlation of the expression of orthologs pair: A randomly chosen abiotic stress induced cucumber *WRKY *genes and a randomly chosen abiotic stress induced *AtWRKY *gene constituted a random WRKY gene pair. This process was repeated a 100 times and produced 100 random WRKY gene pairs. The expression correlation of each of 100 random WRKY gene pair was calculated as described above. Lastly, the average correlation of orthologous WRKY gene pairs and of randomly selected gene pairs was calculated. Student's t-test was used to obtain the statistical significance of the difference in average correlation of the two datasets. The random WRKY genes pairs were obtained using Perl scripts. Pearson correlation and P-values in t-test were calculated by using software R. All programs run on a computer with Ubuntu Linux installed.

### Detection of positive selection

The Amino acid sequence of group 3 *AtWRKY *and *CsWRKY *proteins were used to construct phylogenetic tree respectively, which in turn was used for detecting positive selection. We used PAML4 [66] to analyze codon substitution patterns with a maximum likelihood, implementing a site-specific model. We detected variation in ω values among sites by employing a likelihood ratio test (LRT) between M0 vs. M3 and M7 vs. M8 according to Yang *et al. *[67]. The nodes were considered to have undergone positive selection, if they satisfied the following criteria: (1) an estimate of ω > 1 under M8 (2) sites identified to be under positive selection by Bayes Empirical Bayes (BEB) analysis and (3) a statistically significant LRT.

### Plant materials, growth conditions and treatments

Line 9930, a cucumber typical of northern China, was used throughout the study. Seeds were germinated in pots containing vermiculite, and 3-week old seedlings were used in the following treatments. For dehydration treatment, the plants were carefully pulled out, transferred on to filter paper and allowed to dry. For salinity and cold treatments, seedlings were subjected to a 100 mM NaCl solution or incubated at 4°C, respectively. Above-ground samples for RNA extractions were collected at 0, 0.5, 1, 3, 6, 12 and 24 h after treatment. The roots, stems, leaves, cotyledons of seedlings, female flowers, male flowers and fruits of mature plants were collected separately for RNA isolation and used for tissue-specific expression analysis.

### RNA isolation, clone full-length cDNA, RT-PCR and Real -time PCR analysis

Total RNA was isolated according to Zhang *et al.*, [59]. For cloning the full-length cDNA of *CsWRKY *genes, we first used the EST sequences of cucumber to correct the annotated *CsWRKY *sequence and then used the Fgenesh, a web-base gene prediction method, as a tool to re-annotate all 57 WRKY genes. Subsequently, combined the result of Fgenesh, GLEAN and EVM (GLEAN and EVM were employed to annotate cucumber genome in cucumber genome project), we amplified the full-length sequence of *CsWRKY *coding region (CDS) genes by PCR.

For RT-PCR, the specific primers were designed according to the WRKY gene sequences by Primer 5 software (additional file 8). A cucumber *β-actin *gene (ID: Csa017310), amplified with primers 5'-TCCACGAGACTACCTACAACTC-3' and 5'-GCTCATACGGTCAGCGAT-3', was used as a control. The following program was used for RT-PCR: 94 for 2 min followed by 35 cycles at 94 for 10 s, 55-59 for 10 s and 72 for 25 s, followed by a 2 min extension step at 72. While the number of cycles of PCR for actin gene was set as 23. The PCR products were separated on an agarose gel and quantified using an Imaging System (Bio-Rad, USA). The experiments were repeated three times with independent RNA samples.

The real-time PCR analysis were performed using BIO-RAD CFX96 real-Time PCR system(Bio-Rad, USA) 96 well formats with denaturation at 95°C for 3 min, followed by 40 cycles of denaturation at 95°C for 10 s and annealing/extension at 55 or 60°C for 1 min. Three biological replicates were carried out and triplicate quantitative assays for each replicate were performed on 0.5 μl of each cDNA dilution using TianGen SYBR Green PCR Master mix kit (TianGen Biotech FP202, CHN) according to the manufacturer's protocol. The cucumber *β-actin *gene was used as an internal control. Relative gene expression was calculated according to Jiang *et al.*, [68]. The ΔCT and ΔΔCT were calculated by the formulas ΔCT = CT target - CT reference and ΔΔCT = ΔCT treated sample -ΔCT untreated sample (0 h treatment). The RNA relative amount as selected to evaluate gene expression level as 2-ΔΔCT, which was used for all chart preparations. At the same time, the standard errors of mean among replicates were calculated. All calculations were automatically carried on Bio-Rad CFX Manager (Version1.5.534) of BIO-RAD CFX96. Student's t-test was used to obtain the statistical significance of the difference between treated samples and untreated samples (0 h treatment under abiotic stress). If P-values < 0.01, we considered the WRKY genes as differential expressed genes. The specific primers were designed for WRKY genes and *β-actin *gene used in real time PCR were listed in additional file 9. The data and pictures produced by BIO-RAD CFX96 were presented in additional file 10 and additional file 11, respectively.

## List of abbreviations

RT-PCR: reverse transcription PCR; TF: transcription factor; WDs: WRKY domains; ML: Maximum likelihood; NJ: neighbor-joining; dS: the rate of synonymous substitutions; dN: the rate of non-synonymous substitutions.

## Authors' contributions

JL contributed to RNA extraction, RT-PCR, real-time PCR, bioinformatics analysis and writing of the manuscript. YZ and ZCM helped with the RNA extraction, RNA extraction, RT-PCR, and real-time PCR. HJY contributed to the discussion of the evolution pattern of WRKY genes. XFG and SWH contributed to the discussion and calculation of positive selection of WRKY genes. WJJ and BYX designed the experiments and contributed to revisions of the manuscript. All authors read and approved the final manuscript.

## Supplementary Material

Additional file 1 **A rooted phylogenetic tree representing relationships among WRKY domains of rice, cucumber and *Arabidopsis***. The amino acid sequences of the WRKY domain of rice WRKY (*OsWRKY*), *CsWRKY *and *AtWRKY *proteins were used to reconstruct a phylogenetic tree. The most primitive *Giardia lamblia *WRKY C-terminal domain (*GlWRKY*1C) was used as an outgroup. Group 1 proteins with the suffix 'N' or 'C' indicates the N-terminal WRKY domains or the C-terminal WRKY domains. Stars and black lines represent orthologous WRKY of cucumber and *Arabidopsis*. The tree was constructed by PHYLIP 3.2 and displayed by njplot software. Click here for file

Additional file 2 **putative orthologs of cucumber and *Arabidopsis***. Identified WRKY proteins in cucumber and their putative orthologs in *Arabidopsis *based on phylogenetic studies of WRKY domain sequences. Click here for file

Additional file 3 **Amino acid motif analysis of *CsWRKY *proteins from different groups (or subgroups) and selected group 3 *AtWRKY *proteins**. Motif analysis was performed using Meme 4.0 software. The schematic diagram was obtained by Perl-SVG script and edited in photoshop 7.0. Click here for file

Additional file 4 **The schematic diagram of motifs of WRKY proteins**. The schematic diagram was deserved from Meme 4.0 software. The order of motifs of WRKY proteins in the diagram was automatically generated by Meme software according to scores. Click here for file

Additional file 5 **Comparison of expression pattern of orthologous WRKY pairs under various abiotic stresses**. Available expression data on *AtWRKY *genes from microarray analysis and that of *CsWRKY *genes generated by real-time PCR analysis were compared. Click here for file

Additional file 6 **The expression data for calculating the correlation of orthologs under abiotic stresses**. Expression data of *Arabidopsis *from microarray and of cucumber from Real-time RT-PCR analysis were used to calculate the Pearson correlation of the expression of orthologous WRKY genes pairs under various abiotic stress (at 0, 0.5, 1, 3, 6, 12 and 24 h treatment). Click here for file

Additional file 7 **The GenBank accession numbers of WRKY protein sequences used in the manuscript**. GenBank accession numbers of WRKY protein were from NCBI or PFAM database. Click here for file

Additional file 8 **The primer sequences used for RT-PCR amplification of 48 *CsWRKY *genes**. The specific primers were designed according to the WRKY gene sequences by Primer 5 software. Click here for file

Additional file 9 **The primer sequences used for real-time PCR of stress-responsive and group 3 *CsWRKY *genes**. The specific primers were designed according to the WRKY gene sequences by Primer 5 software. Click here for file

Additional file 10 **The expression patterns of stress-inducible *CsWRKY *genes were shown by real-time PCR analyses under three different abiotic stresses**. Expression of stress-inducible *CsWRKY *genes were shown by real-time PCR analyses under three different abiotic stresses. The pictures of the first column, the second column and the third column indicated the expression pattern under cold treatment, drought treatment and salt treatment respectively. For each picture, the y-axis indicated the relative fold of treatment to control and x-axis indicate the time under treatment. (A),*CsWRKY2; *(B),*CsWRKY18*; (C),*CsWRKY21; *(D),*CsWRKY40; *(E),*CsWRKY46*. This is the originally pictures produced by Bio-Rad CFX manager software automatically. Click here for file

Additional file 11 **The Ct-values and standard deviation for the real time RT-PCR of CsWRKY genes**. The Ct-value and standard deviation of CsWRKY genes and their corresponding actin control under different treatments. Click here for file

## References

[B1] Eulgem T Rushton PJ Robatzek S Somssich IE The WRKY superfamily of plant transcription factors Trends Plant Sci 2000 5 199 206 10.1016/S1360-1385(00)01600-9 10785665

[B2] Rushton PJ Macdonald H Huttly AK Lazarus CM Hooley R Members of a new family of DNA-binding proteins bind to a conserved cis-element in the promoters of a-Amy2 genes Plant Mol Biol 1995 29 691 702 10.1007/BF00041160 8541496

[B3] Sun C Palmqvist S Olsson H Boren M Ahlandsberg S Jansson C A novel WRKY transcription factor, SUSIBA2, participates in sugar signaling in barley by binding to the sugarresponsive elements of the iso1 promoter Plant Cell 2003 15 2076 2092 10.1105/tpc.014597 12953112 PMC181332

[B4] Kazuhiko Y Takanori K Makoto I Masaru T Tomoko Y Takashi Y Masaaki A Eiko S Takayoshi M Yasuko T Nobuhiro H Takaho T Mikako S Akiko T Motoaki S Kazuo S Shigeyuki Y Solution Structure of an *Arabidopsis *WRKY DNA Binding Domain Plant Cell 2005 17 944 956 10.1105/tpc.104.026435 15705956 PMC1069710

[B5] Ishiguro S Nakamura K Characterization of a cDNA encoding a novel DNA-binding protein, SPF1, that recognizes SP8 sequences in the 59 upstream regions of genes coding for sporamin and b-amylase from sweet potato Mol Gen Genet 1994 244 563 571 7969025 10.1007/BF00282746

[B6] Rushton PJ Torres JT Parniske M Wernert P Hahlbrock K Somssich IE Interaction of elicitor-induced DNA-binding proteins with elicitor response elements in the promoters of parsley PR1 genes EMBO J 1996 15 5690 5700 8896462 PMC452313

[B7] Kim DJ Smith SM Leaver CJ A cDNA encoding a putative SPF1-type DNA-binding protein from cucumber Gene 1997 185 265 269 10.1016/S0378-1119(96)00665-8 9055825

[B8] Dellagi A Heilbronn J Avrova A Montesano M Palva ET Stewart HE Toth IK Cooke D Lyon G Birch P A potato gene encoding a WRKY-like transcription factor is induced in interactions with Erwinia carotovora subsp atroseptica and Phytophthora infestans and is coregulated with class I endochitinase expression Mol Plant-Microbe Interact 2000 13 1092 1101 10.1094/MPMI.2000.13.10.1092 11043470

[B9] Huang T Duman JG Cloning and characterization of a thermal hysteresis (antifreeze) protein with DNA-binding activity from winter bittersweet nightshade, *Solanum dulcamara* Plant Mol Biol 2002 48 339 350 10.1023/A:1014062714786 11905961

[B10] Pnueli L Hallak HE Rozenberg M Cohen M Goloubinoff P Kaplan A Mittler R Molecular and biochemical mechanisms associated with dormancy and drought tolerance in the desert legume *Retama raetam* Plant J 2002 31 319 330 10.1046/j.1365-313X.2002.01364.x 12164811

[B11] Ulker B Somssich IE WRKY transcription factors: from DNA binding towards biological function Curr Opin Plant Biol 2004 7 491 498 10.1016/j.pbi.2004.07.012 15337090

[B12] Mantri NL Ford R Coram TE Pang EC Transcriptional profiling of chickpea genes differentially regulated in response to highsalinity, cold and drought BMC Genomics 2007 8 303 10.1186/1471-2164-8-303 17764573 PMC2025592

[B13] Kato N Dubouzet E Kokabu Y Yoshida S Taniguchi Y Dubouzet JG Yazaki K Sato F Identification of a WRKY protein as a transcriptional regulator of benzylisoquinoline alkaloid biosynthesis in *Coptis japonica* Plant Cell Physiol 2007 48 8 18 17132631 10.1093/pcp/pcl041

[B14] Marchive C Mzid R Deluc L Barrieu F Pirrello J Gauthier A Corio-Costet A Regad F Cailleteau B Hamdi S Lauvergeat V Isolation and characterization of a *Vitis vinifera *transcription factor, VvWRKY1, and its effect on responses to fungal pathogens in transgenic tobacco plants J Exp Bot 2007 58 1999 2010 10.1093/jxb/erm062 17456504

[B15] Zhou QY Tian AG Zou HF Xie ZM Lei G Huang J Wang CM Wang HW Zhang JS Chen SY Soybean WRKY-type transcription factor genes, GmWRKY13, GmWRKY21, and GmWRKY54, confer differential tolerance to abiotic stress in transgenic *Arabidopsis *plants Plant Biotechnol J 2008 6 486 503 10.1111/j.1467-7652.2008.00336.x 18384508

[B16] Liu JJ Ekramoddoullah AK Identification and characterization of the WRKY transcription factor family in Pinus monticola Genome 2009 52 77 88 10.1139/G08-106 19132074

[B17] Wu KL Guo ZJ Wang HH Li J The WRKY family of transcription factors in rice and *Arabidopsis *and their origins DNA Research 2005 12 9 26 10.1093/dnares/12.1.9 16106749

[B18] Ramamoorthy R Jiang SY Kumar N Venkatesh PN Ramachandran S A comprehensive transcriptional profiling of the WRKY gene family in rice under various abiotic and phytohormone treatments Plant Cell Physiol 2008 49 865 879 10.1093/pcp/pcn061 18413358

[B19] Dong J Chen C Chen Z Expression profiles of the *Arabidopsis *WRKY gene superfamily during plant defense response Plant Mol Biol 2003 51 21 37 10.1023/A:1020780022549 12602888

[B20] Xu X Chen C Fan B Chen Z Physical and functional interactions between pathogen-induced *Arabidopsis *WRKY18, WRKY40, and WRKY60 transcription factors Plant Cell 2006 18 1310 1326 10.1105/tpc.105.037523 16603654 PMC1456877

[B21] Li J Brader G Kariola T Palva T WRKY70 modulates the selection of signaling pathways in plant defense Plant J 2006 46 477 491 10.1111/j.1365-313X.2006.02712.x 16623907

[B22] Oh SK Yi SY Yu SH Moon JS Park JM Choi D CaWRKY2, a chili pepper transcription factor, is rapidly induced by incompatible plant pathogens Mol Cells 2006 22 58 64 16951551

[B23] Zheng Z Mosher SL Fan B Klessig DF Chen Z Functional analysis of *Arabidopsis *WRKY25 transcription factor in plant defense against Pseudomonas syringae BMC Plant Biol 2007 7 2 10.1186/1471-2229-7-2 17214894 PMC1780049

[B24] Zheng Z Qamar SA Chen Z Mengiste T *Arabidopsis *WRKY33 transcription factor is required for resistance to necrotrophic fungal pathogens Plant J 2006 48 592 605 10.1111/j.1365-313X.2006.02901.x 17059405

[B25] Beyer K Binder A Boller T Colling M Identification of potato genes induced during colonization by Phytophthora infestans Mol Plant Pathol 2001 2 125 134 10.1046/j.1364-3703.2001.00059.x 20573000

[B26] Kalde M Barth M Somssich IE Lippok B Members of the *Arabidopsis *WRKY group III transcription factors are part of different plant defense signaling pathways Mol Plant-Microbe Interact 2003 16 295 305 10.1094/MPMI.2003.16.4.295 12744458

[B27] Knoth C Ringler J Dangl JL Eulgem T *Arabidopsis *WRKY70 is required for full RPP4-mediated disease resistance and basal defense against *Hyaloperonospora parasitica* Mol Plant-Microbe Interact 2007 20 120 128 10.1094/MPMI-20-2-0120 17313163

[B28] Johnson SC Kolevski B Smyth DR *Transparent testa glabra2*, a trichome and seed coat development gene of *Arabidopsis*, encodes a WRKY transcription factor Plant Cell 2002 14 1359 1375 10.1105/tpc.001404 12084832 PMC150785

[B29] Lagace M Matton DP Characterization of a WRKY transcription factor expressed in late torpedo-stage embryos of *Solanum chacoense* Planta 2004 219 185 189 10.1007/s00425-004-1253-2 15045588

[B30] Robatzek S Somssich IE Targets of AtWRKY6 regulation during plant senescence and pathogen defense Genes Dev 2002 16 1139 1149 10.1101/gad.222702 12000796 PMC186251

[B31] Zhang ZL Xie Z Zou X Casaretto J David TH Zhen QJ A rice WRKY gene encodes a transcriptional repressor of the gibberellin signaling pathway in aleurone cells Plant Physiol 2004 134 1500 1513 10.1104/pp.103.034967 15047897 PMC419826

[B32] Zou X Seemann JR Neuman D Shen QJ A WRKY gene from creosote bush encodes an activator of the abscisic acid signaling pathway J Biol Chem 2004 279 55770 55779 10.1074/jbc.M408536200 15504732

[B33] Xie Z Zhang ZL Zou X Yang G Komatsu S Shen QJ Interactions of two abscisic-acid induced WRKY genes in repressing gibberellin signaling in aleurone cells Plant J 2006 46 231 242 10.1111/j.1365-313X.2006.02694.x 16623886

[B34] Du L Chen Z Identification of genes encoding receptorlike protein kinases as possible targets of pathogen- and salicylic acid-induced WRKY DNA-binding proteins in *Arabidopsis* Plant J 2002 24 837 847 10.1046/j.1365-313x.2000.00923.x 11135117

[B35] Karam BS Rhonda CF Luis OS Transcription factors in plant defense and stress response Curr Opin Plant Biol 2002 5 430 436 10.1016/S1369-5266(02)00289-3 12183182

[B36] Motoaki S Mari NJ Ishida TN Miki F Youko O Asako K Maiko N Akiko E Tetsuya S Masakazu S Kenji A Teruaki T Kazuko YS Piero C Jun K Yoshihide H Kazuo S Monitoring the expression profiles of 7000 *Arabidopsis *genes under drought, cold and high-salinity stresses using a full-length cDNA microarray Plant J 2002 31 279 292 10.1046/j.1365-313X.2002.01359.x 12164808

[B37] Kilian J Whitehead D Horak J Wanke D Weinl S Batistic O D'Angelo C Bornberg-Bauer E Kudla J Harter K The AtGenExpress global stress expression data set: protocols, evaluation and model data analysis of UV-B light, drought and cold stress responses Plant J 2007 50 347 363 10.1111/j.1365-313X.2007.03052.x 17376166

[B38] Mare C Mazzucotelli E Crosatti C Francia E Stanca AM Cattivelli L Hv-WRKY38: a new transcription factor involved in cold- and drought-response in barley Plant Mol Biol 2004 55 399 416 10.1007/s11103-004-0906-7 15604689

[B39] Liu SQ Xu L Jia ZQ Xu Y Yang Q Fei ZJ Lu XY Chen HM Huang SW Genetic association of ETHYLENE-INSENSITIVE3-like sequence with the sex-determining M locus in cucumber (*Cucumis sativus L*.) Theor Appl Genet 2004 117 927 933 10.1007/s00122-008-0832-1 18629467

[B40] Huang SW Li RQ Zhang ZH Li L Gu XF Fan W Lucas WJ Wang XW Xie BY Ni PX Ren YY Zhu HM Li J Lin K Jin WW Fei ZJ Li GC Staub J Kilian A Vossen EAGV Wu Y Guo J He J Jia ZQ Ren Y Tian G Lu Y Ruan J Qian WB Wang MW The genome of the cucumber, *Cucumis sativus L* Nature Genetic 2009 475 1 7 10.1038/ng.475 19881527

[B41] Ross CA Liu Y Shen QJ The WRKY gene family in rice (*Oryza sativa*) J Integr Plant Biol 2007 49 827 842 10.1111/j.1744-7909.2007.00504.x

[B42] Rossberg M Theres K Acarkan A Herrero R Schmitt T Schumacher K Schmitz G Schmidt R Comparative sequence analysis reveals extensive microcolinearity in the lateral suppressor regions of the tomato, *Arabidopsis*, and Capsella genomes Plant Cell 2001 13 979 988 11283350 10.1105/tpc.13.4.979 PMC135537

[B43] Schauser L Wieloch W Stougaard J Evolution of NIN-like proteins in *Arabidopsis*, rice, and *Lotus japonicus* J Mol Evol 2005 60 229 237 10.1007/s00239-004-0144-2 15785851

[B44] Zhang YJ Wang LJ The WRKY transcription factor superfamily: its origin in eukaryotes and expansion in plants BMC Evolutionary Biology 2005 5 1 10.1186/1471-2148-5-1 15629062 PMC544883

[B45] Blanc G Hokamp K Wolfe KH A recent polyploidy superimposed on older large-scale duplications in the *Arabidopsis *genome Genome Res 2003 13 137 144 10.1101/gr.751803 12566392 PMC420368

[B46] Cannon SB Mitra A Baumgarten A Young ND May G The roles of segmental and tandem gene duplication in the evolution of large gene families in *Arabidopsis thaliana* BMC Plant Biol 2004 4 10 10.1186/1471-2229-4-10 15171794 PMC446195

[B47] Taylor JS Raes J Duplication and divergence: The evolution of new genes and old ideas Annu Rev Genet 2004 38 615 643 10.1146/annurev.genet.38.072902.092831 15568988

[B48] Jaillon O Aury JM Noel B Policriti A Clepet C Casagrande A Choisne N Aubourg S Vitulo N Jubin C Vezzi A Legeai F Hugueney P Dasilva C Horner D Mica E Jublot D Poulain J Bruyere C Billault A Segurens B Gouyvenoux M Ugarte E Cattonaro F Anthouard V Vico V Del Fabbro C Alaux M Di Gaspero G Dumas V The grapevine genome sequence suggests ancestral hexaploidization in major angiosperm phyla Nature 2007 449 463 467 10.1038/nature06148 17721507

[B49] S. Berri P Abbruscato O Faivre-Rampant AC Brasileiro I Fumasoni K Satoh S Kikuchi L Mizzi P Morandini ME Pe P Piffanelli P Characterization of WRKY co-regulatory networks in rice and *Arabidopsis* BMC Plant Biol 2009 9 120 10.1186/1471-2229-9-120 19772648 PMC2761919

[B50] Li L Stoeckert CJ Jr Roos DS OrthoMCL: identification of ortholog groups for eukaryotic genomes Genome Res 2003 13 2178 2189 10.1101/gr.1224503 12952885 PMC403725

[B51] Chen F Mackey AJ Vermunt JK Roos DS Assessing performance of orthology detection strategies applied to eukaryotic genomes PLoS ONE 2007 2 e383 10.1371/journal.pone.0000383 17440619 PMC1849888

[B52] Mangelsen E Kilian J Berendzen KW Kolukisaoglu H Harter K Jansson C Wanke D Phylogenetic and comparative gene expression analysis of barley (*Hordeum vulgare*) WRKY transcription factor family reveals putatively retained functions between monocots and dicots BMC Genomics 2008 9 194 10.1186/1471-2164-9-194 18442363 PMC2390551

[B53] Blanc G Wolfe KH Functional divergence of duplicated genes formed by polyploidy during *Arabidopsis *evolution Plant Cell 2004 16 1679 1691 10.1105/tpc.021410 15208398 PMC514153

[B54] Zhang J Evolution by gene duplication--an update Trends Ecol Evol 2003 18 292 298 10.1016/S0169-5347(03)00033-8

[B55] The *Arabidopsis *Information Resource (TAIR) http://www.arabidopsis.org/

[B56] Rice Genome Annotation Project http://rice.plantbiology.msu.edu/index.shtml

[B57] The Pfam database of protein domains and HMMs http://pfam.jouy.inra.fr/

[B58] Cucumber Genome DataBase http://cucumber.genomics.org.cn/page/cucumber/index.jsp

[B59] Thompson JD Gibson TJ Plewniak F Jeanmougin F Higgins DG The CLUSTAL_X windows interface: flexible strategies for multiple sequence alignment aided by quality analysis tools Nucleic Acids Res 1997 25 4876 4882 10.1093/nar/25.24.4876 9396791 PMC147148

[B60] Tamura K Dudley J Nei M Kumar S MEGA4: Molecular Evolutionary Genetics Analysis (MEGA) software version 4.0 Molecular Biology and Evolution 2007 24 1596 1599 10.1093/molbev/msm092 17488738

[B61] Felsenstein J PHYLIP--Phylogeny Inference Package (Version 3.2) Cladistics 1989 5 164 166

[B62] Zhang GY Chen M Chen XP Xu ZS Guan S Li LC Li AL Guo JM Mao L Ma YZ Phylogeny, gene structures, and expression patterns of the ERF gene family in soybean (*Glycine max L*.) J Exp Bot 2008 59 4095 4107 10.1093/jxb/ern248 18832187 PMC2639015

[B63] Bailey TL Williams N Misleh C Li WW MEME: discovering and analyzing DNA and protein sequence motifs Nucleic Acids Res 2006 34 W369 W373 10.1093/nar/gkl198 16845028 PMC1538909

[B64] Jorge I Ribichich Karina F Dezar Carlos A Chan Raquel L Expression analyses indicate the involvement of sunflower WRKY transcription factors in stress responses, and phylogenetic reconstructions reveal the existence of a novel clade in the Asteraceae Plant Science 2010 178 398 410 10.1016/j.plantsci.2010.02.008

[B65] Weigel World Database http://www.weigelworld.org/resources/microarray/AtGenExpress/

[B66] Yang Z PAML 4: phylogenetic analysis by maximum likelihood Mol Biol Evol 2007 24 1586 1591 10.1093/molbev/msm088 17483113

[B67] Yang Z Gu S Wang X Li W Tang Z Xu C Molecular evolution of the CPP-like gene family in plants: Insights from comparative genomics of *Arabidopsis *and rice J Mol Evol 2008 67 266 277 10.1007/s00239-008-9143-z 18696028

[B68] Jiang SY Bachmann D La H Ma Z Venkatesh PN Ramamoorthy R Ramachandran S Ds insertion mutagenesis as an efficient tool to produce diverse variations for rice breeding Plant Mol Biol 2007 65 385 402 10.1007/s11103-007-9233-0 17891459

