# Supplementary figures and images for: Genome-wide analysis of WRKY gene family in Cucumis sativus
Source: BMC Genomics. 2011 Sep 28;12:471. doi: 10.1186/1471-2164-12-471 (PMC3191544; doi:10.1186/1471-2164-12-471)

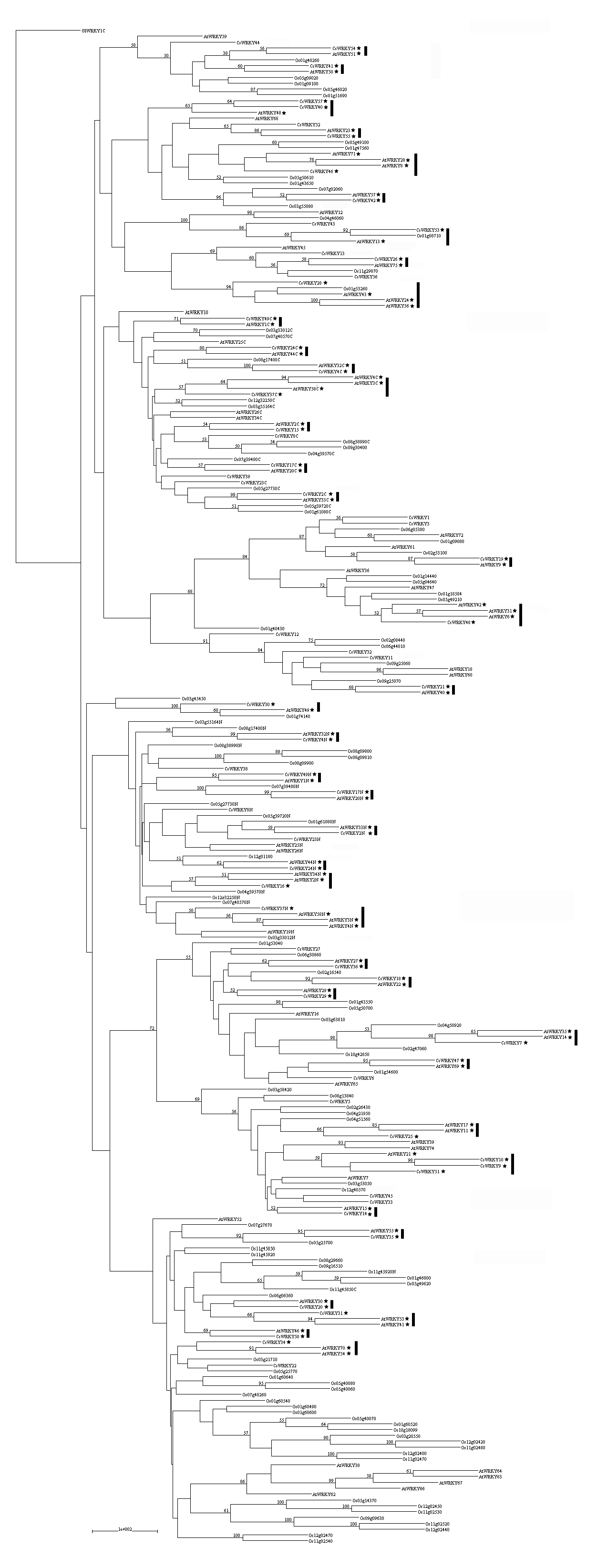

Supplement: Additional file 1 — A rooted phylogenetic tree representing relationships among WRKY domains of rice, cucumber and Arabidopsis. The amino acid sequences of the WRKY domain of rice WRKY (OsWRKY), CsWRKY and AtWRKY proteins were used to reconstruct a phylogenetic tree. The most primitive Giardia lamblia WRKY C-terminal domain (GlWRKY1C) was used as an outgroup. Group 1 proteins with the suffix 'N' or 'C' indicates the N-terminal WRKY domains or the C-terminal WRKY domains. Stars and black lines represent orthologous WRKY of cucumber and Arabidopsis. The tree was constructed by PHYLIP 3.2 and displayed by njplot software. [file 1471-2164-12-471-S1.TIFF]

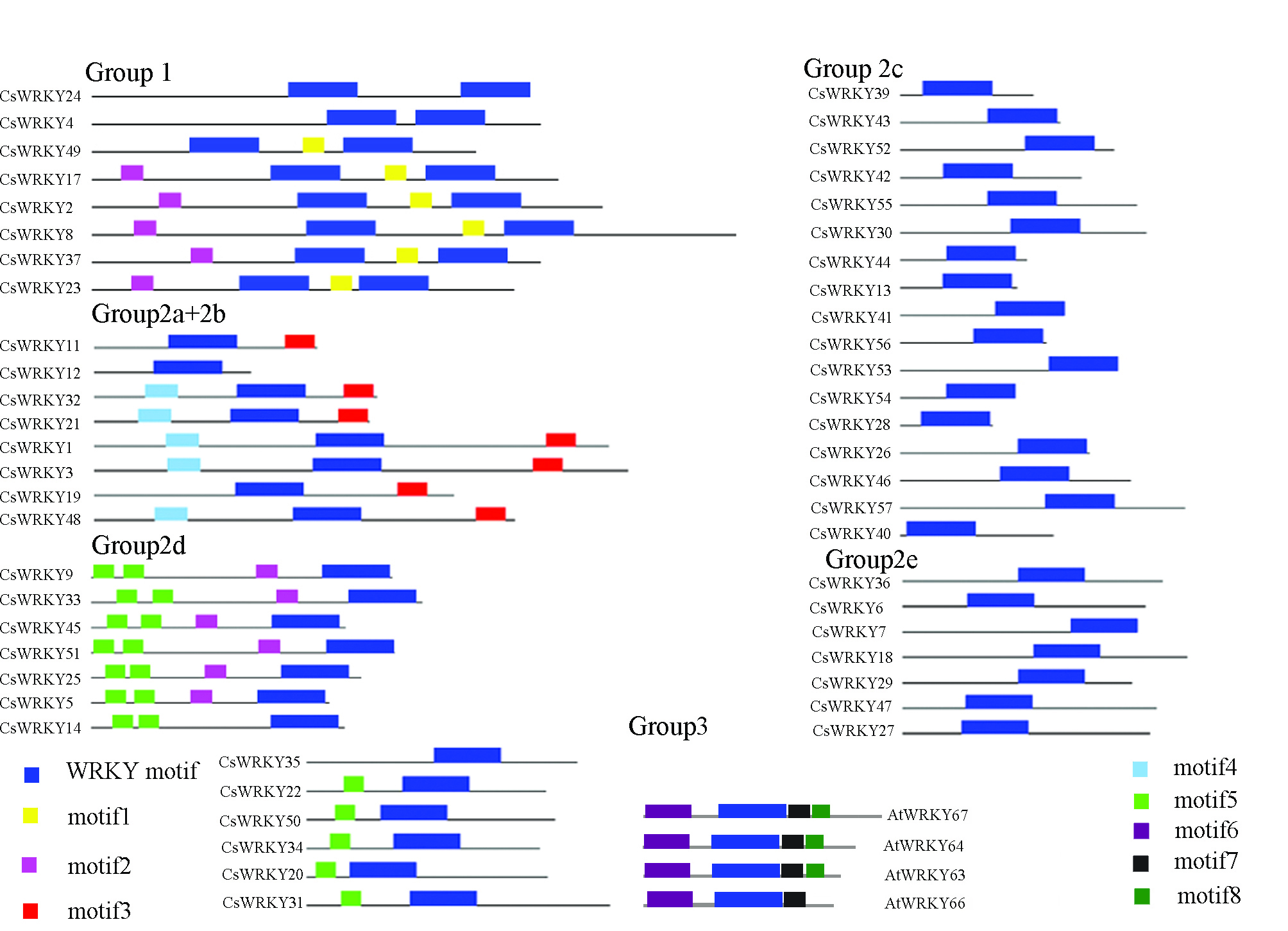

Supplement: Additional file 3 — Amino acid motif analysis of CsWRKY proteins from different groups (or subgroups) and selected group 3 AtWRKY proteins. Motif analysis was performed using Meme 4.0 software. The schematic diagram was obtained by Perl-SVG script and edited in photoshop 7.0. [file 1471-2164-12-471-S3.JPEG]

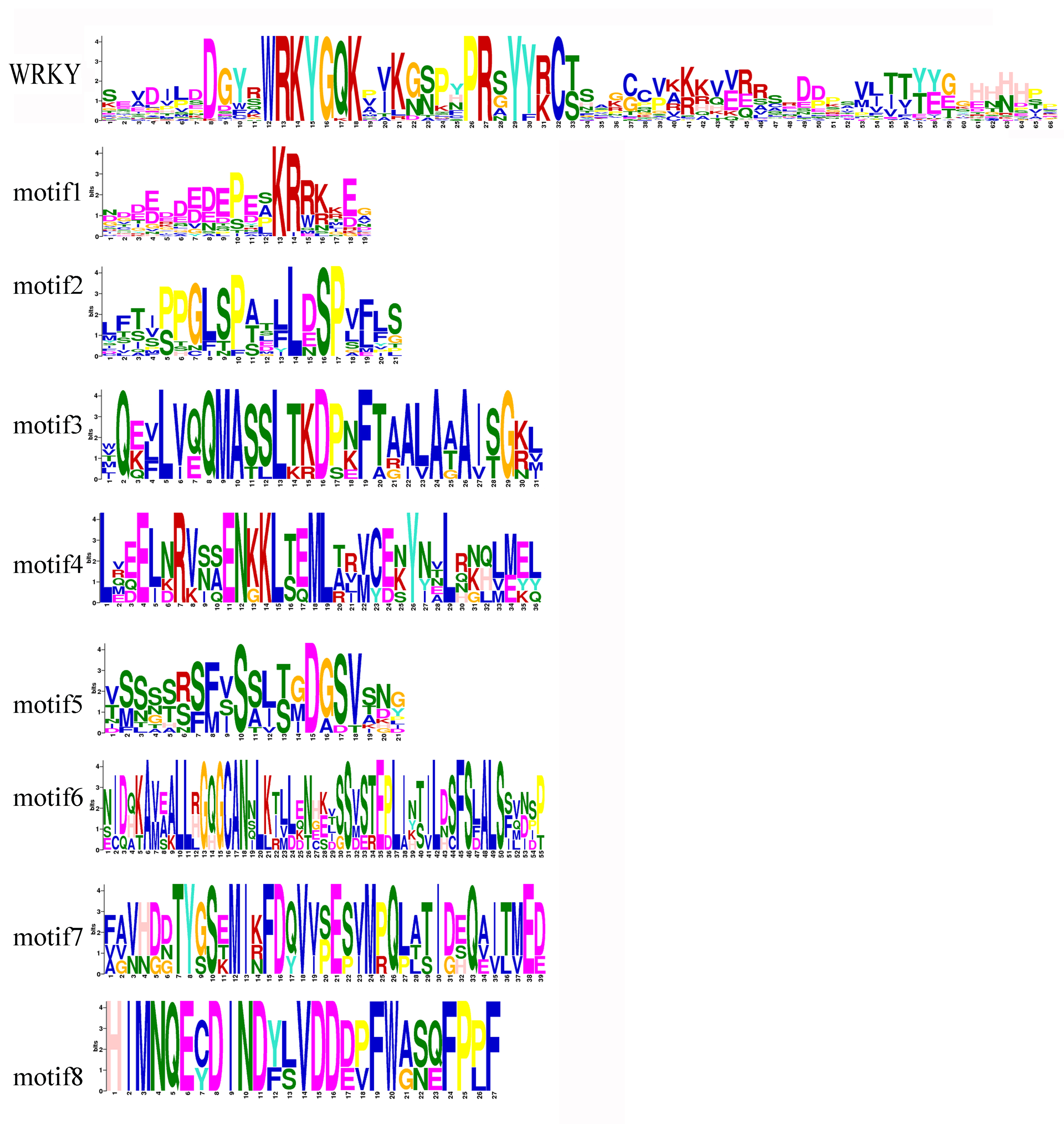

Supplement: Additional file 4 — The schematic diagram of motifs of WRKY proteins. The schematic diagram was deserved from Meme 4.0 software. The order of motifs of WRKY proteins in the diagram was automatically generated by Meme software according to scores. [file 1471-2164-12-471-S4.JPEG]

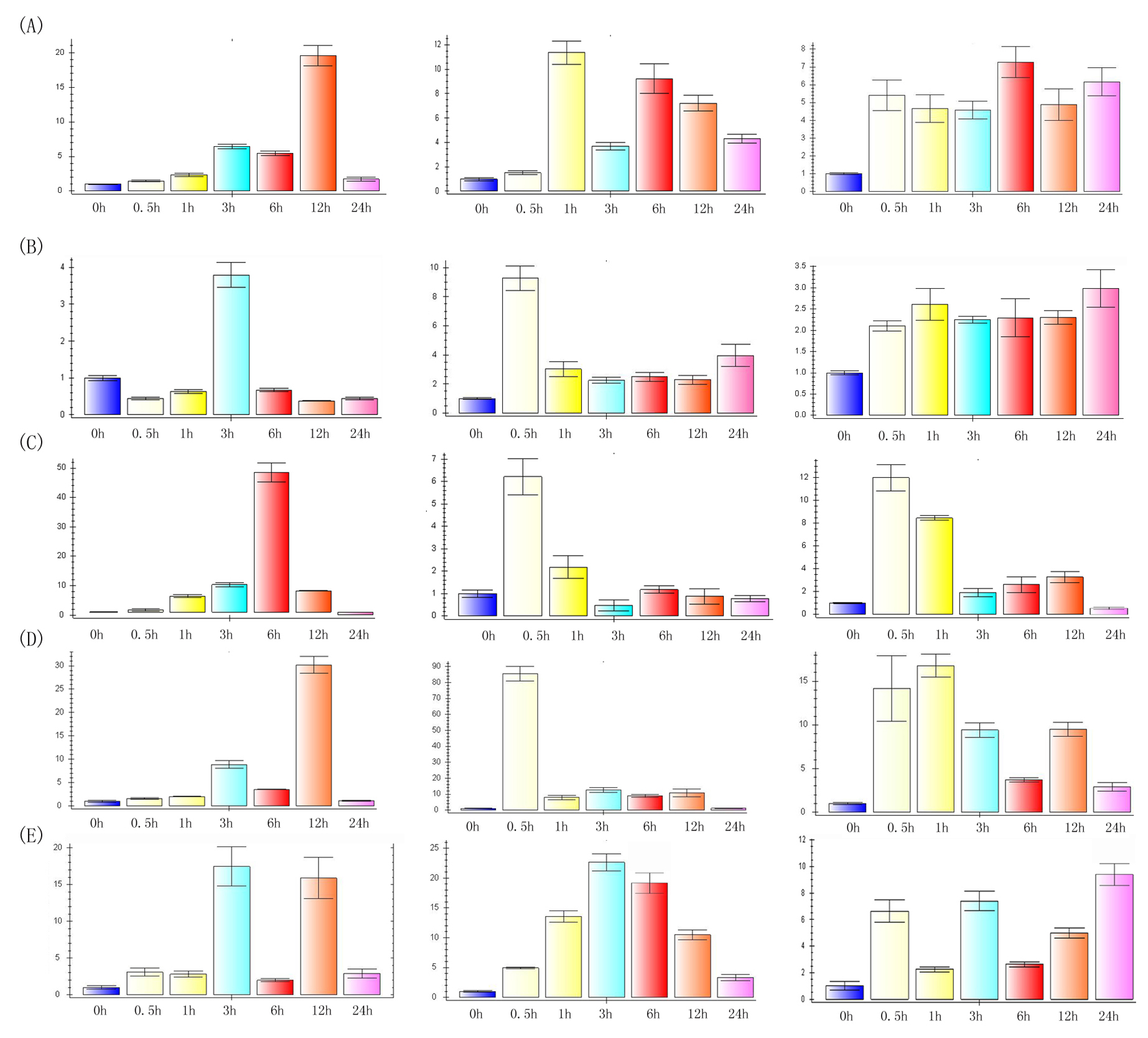

Supplement: Additional file 10 — The expression patterns of stress-inducible CsWRKY genes were shown by real-time PCR analyses under three different abiotic stresses. Expression of stress-inducible CsWRKY genes were shown by real-time PCR analyses under three different abiotic stresses. The pictures of the first column, the second column and the third column indicated the expression pattern under cold treatment, drought treatment and salt treatment respectively. For each picture, the y-axis indicated the relative fold of treatment to control and x-axis indicate the time under treatment. (A),CsWRKY2; (B),CsWRKY18; (C),CsWRKY21; (D),CsWRKY40; (E),CsWRKY46. This is the originally pictures produced by Bio-Rad CFX manager software automatically. [file 1471-2164-12-471-S10.JPEG]
